# A general strategy to control antibody specificity against targets showing molecular and biological similarity: *Salmonella* case study

**DOI:** 10.1038/s41598-020-75285-1

**Published:** 2020-10-28

**Authors:** Riccardo Marega, N. Desroche, A.-C. Huet, M. Paulus, C. Suarez Pantaleon, D. Larose, P. Arbault, P. Delahaut, N. Gillard

**Affiliations:** 1 CER Groupe, Analytical Laboratory, Rue du Point du Jour 8, 6900 Marloie, Belgium; 2 NEXIDIA S.A.S., Rue de Mayence 15, 21000 Dijon, France; 3 Unisensor S.A., Rue Louis Plescia 8, 4102 Ougrée (Liège), Belgium

**Keywords:** Antibody generation, ELISA

## Abstract

The control of antibody specificity plays pivotal roles in key technological fields such as diagnostics and therapeutics. During the development of immunoassays (IAs) for the biosensing of pathogens in food matrices, we have found a way to rationalize and control the specificity of polyclonal antibodies (sera) for a complex analytical target (the *Salmonella* genus), in terms of number of analytes (*Salmonella* species) and potential cross-reactivity with similar analytes (other bacteria strains). Indeed, the biosensing of *Salmonella* required the development of sera and serum mixtures displaying homogeneous specificity for a large set of strains showing broad biochemical variety (54 *Salmonella* serovars tested in this study), which partially overlaps with the molecular features of other class of bacteria (like specific serogroups of *E. coli*). To achieve a trade-off between specificity harmonisation and maximization, we have developed a strategy based on the conversion of the specificity profiles of individual sera in to numerical descriptors, which allow predicting the capacity of serum mixtures to detect multiple bacteria strains. This approach does not imply laborious purification steps and results advantageous for process scaling-up, and may help in the customization of the specificity profiles of antibodies needed for diagnostic and therapeutic applications such as multi-analyte detection and recombinant antibody engineering, respectively.

## Introduction

In many biotechnological and biomedical areas, there is a large demand for deployment of molecular structures able to act as receptors for different class of ligands. In the therapeutic field, this means the development of macromolecules, such as antibodies (Abs) or other biomolecules, able to bind endogenous or exogenous epitopes (e.g. cell-membrane proteins or toxins, respectively), in order to elicit a therapeutic action^1^.

In the diagnostic field, it means the realisation of receptors able to bind analytes that are important for the monitoring of human and animal health^2^, food safety^3,4^, and environmental pollution^5–7^.

As receptors for diagnostic purposes, several class of molecules have been identified and developed^8^, such as Abs^9^, aptamers^10,11^, and molecularly imprinted polymers^12,13^, among others.

The possibility of modulating Abs sensitivity and specificity upon mixing multiple constituents was long ago explored by Ehrlich et al*.*, which were able to strongly increase the analytical sensitivity of ELISAs against the human chorionic gonadotropin^14^. In their studies, different monoclonal antibodies (mAbs) were mixed, resulting in cooperation to form circular complexes between mAbs and antigen, and thus sensitivity and specificity enhancement^15–17^. These and other studies paved the way for the concept of using oligoclonal antibodies (oAbs, mixtures of selected mAbs), which are nowadays commercialized for diagnostic applications (e.g. oligoclonal anti-species labelled antibodies)^18^, and under consideration for therapeutic applications (e.g. oligoclonal mAbs/recombinant pAbs mixtures)^19–21^.

One particular domain where specify modulation of Abs results critical and challenging is food analysis, due to the need of targeting multiple analytes at a same time (e.g. multiple residue veterinary drugs^22^ or multiple bacteria strains^23^) and to the inherent complexity of the food matrices^24,25^. For instance, there is a continuous interest in developing diagnostic assays for the detection of the bacteria strains belonging to the *Salmonella* genus^26,27^, linked with salmonellosis, one of the main bacterial gastroenteric syndromes in industrialized countries, and typhoid fevers. The genus *Salmonella* comprises two species (*S. enterica* and *S. bongori*), the species *S. enterica* itself being divided into 6 subspecies (*enterica*, *salamae*, *arizonae*, *diarizonae*, *houtenae* and *indica*) based on phenotypic criteria.

Serology, based on the characterization of somatic (O) and flagellar (H) antigens, allows the classification of subspecies into serotypes; those belonging to the subspecies enterica are assigned a name frequently corresponding to a geographical place (Dublin, Minnesota), the others are designated by their antigenic formula^28^.

During the development of immunoassays (IAs) for the multiplexed detection of *Salmonella* in food matrices, we have studied how to control sera specificity toward increasingly complex analytes (bacteria strains) in terms of number of analytes and analyte similarity. Furthermore, this multi-analyte system (*Salmonella*) requires sera displaying harmonised responses toward very different targets (*Salmonella* serovars), while keeping low cross reactivity for other pathogens (e.g. *E. coli* and *Citrobacter sp*). To achieve a trade-off between these challenging specificity requirements, we have developed a multistep strategy that allows managing serum mixtures composition (type, number of sera, and their volumetric ratios), which maximize and harmonize the specificity towards the selected targets (the *Salmonella* strains used in this study), and minimize the cross-reactivity against the unwanted ones, with the minimal experimental effort.

## Results and discussion

**Scheme 1:**
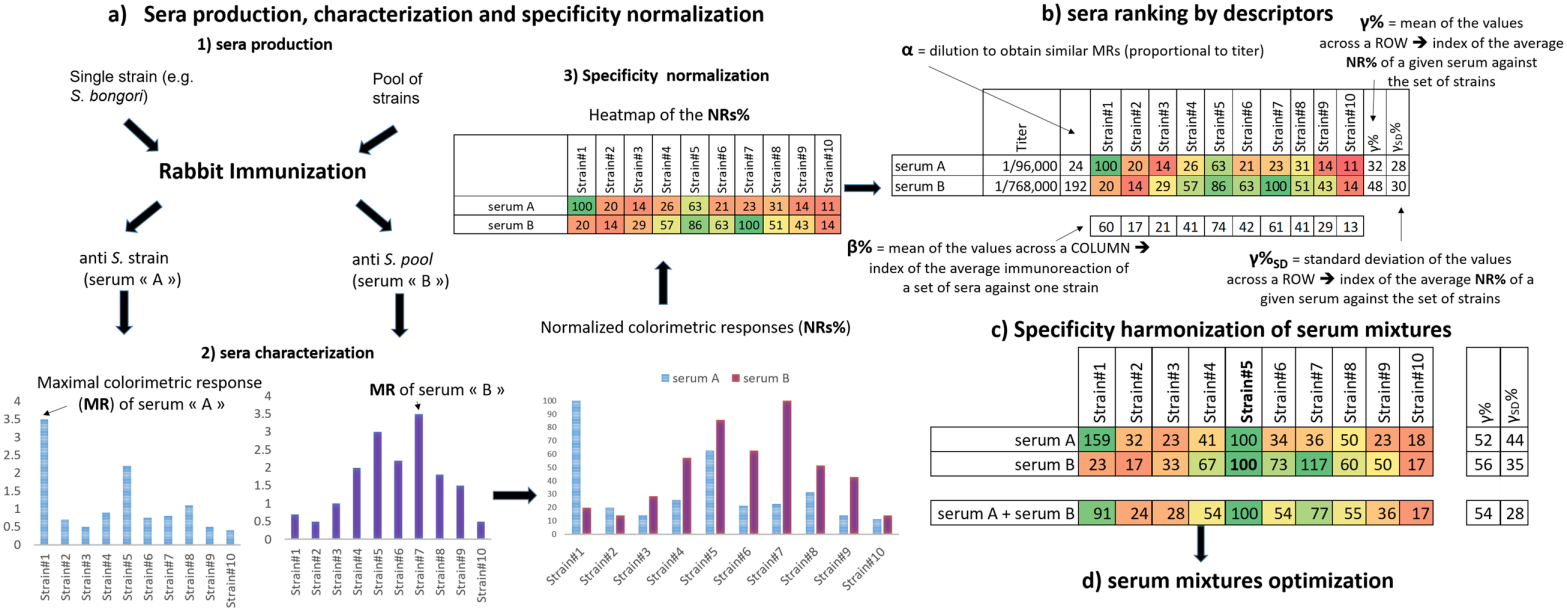
General workflow for the design and preparation of serum mixtures with custom specificity profiles. (**a**) Sera production by rabbit immunization with individual or pooled *Salmonella* strains, and the preliminary characterization by colorimetric indirect ELISA. This yields an ensemble of specificity profiles, one per serum, which are expressed after a simple data treatment as heatmaps of the normalized colorimetric responses (*NRs%).* (**b**) Conversion of the titer values and colorimetric dataset into descriptors (*α*, *β%*, *γ%* ± *γ%*_*SD*_), allows ranking the sera. (**c**) Upon re-calculating the *NRs%* against a common reference strain, the specificity profiles of a given mixture can be predicted and experimentally verified. (**d**) Final step of serum mixtures optimization aiming at the simplification of their composition and fine-tuning of their specificity profiles. The values presented in this scheme are just explanatory of the general workflow, and thus they do not correspond to the actual dataset.

The general workflow of this study is based on the four stages of development summarized in Scheme 1, such as: a) sera production (by immunisation against *Salmonella* strains) and their characterization (specificity normalization by indirect colorimetric ELISA); b) The use of different descriptors to rank the sera; c) Specificity harmonization of serum mixtures (by rational mixing of selected sera); and d) Serum mixtures optimization, in terms of number of sera and fine tuning of the specificity profiles.

### Sera production, characterization and specificity normalization

As a way to produce sera and evaluate their relative specificity, we used heat-inactivated bacterial cells belonging to the genus *Salmonella* (54 serovars), the family of Enterobacteriaceae or belonging to other genus or species such as *Staphylococcus* and *Pseudomonas sp* (Supporting Information S1).

All of the *Salmonella* strains used as immunogens were tested during titer and specificity assessments, while some strains were only used during the specificity determinations. Those strains used as immunogens were either used individually (see entries 29–54 in Supporting Information S1), or mixed together to form pools of *Salmonella* strains (pools; see entries 55–61 in Supporting Information S1), based on mixtures of strains that are relevant in food analysis, and by considering their antigenic properties (*pool#1a*, *pool#1b*, *pool#1c*, *pool#2*, *pool#3*, *pool#4a* and *pool#4b*). The rationale is to exploit rabbits immune repertoires to generate a library of reagents (the pAbs in the hyperimmune sera), and to verify which one of the following scenarios is the most suitable to address the problem of custom specificity profiles generation: (1) use of individual sera obtained upon immunization against multiple strains (specificity modulation via “serendipitous/stochastic” means); (2) use of multiple sera obtained by immunization against individual strains (specificity modulation via a rational and linear approach): or (3) combinations between sera belonging to the two aforementioned categories, where elements from (1) will serve as a “base” and elements from (2) as further constituents for finer specificity tuning against selected strains. In this, study 21 different immunogens and two biological replicates were used, yielding 14 sera against *Salmonella* pools and 28 sera against individual *Salmonella* strains. The strains used as immunogens were injected into rabbits by intradermal route, since this administration path resulted more efficient in eliciting higher (p < 0.01) and more homogeneous titres compared to the intravenous route (Supporting Information S2).

**Figure 1:**
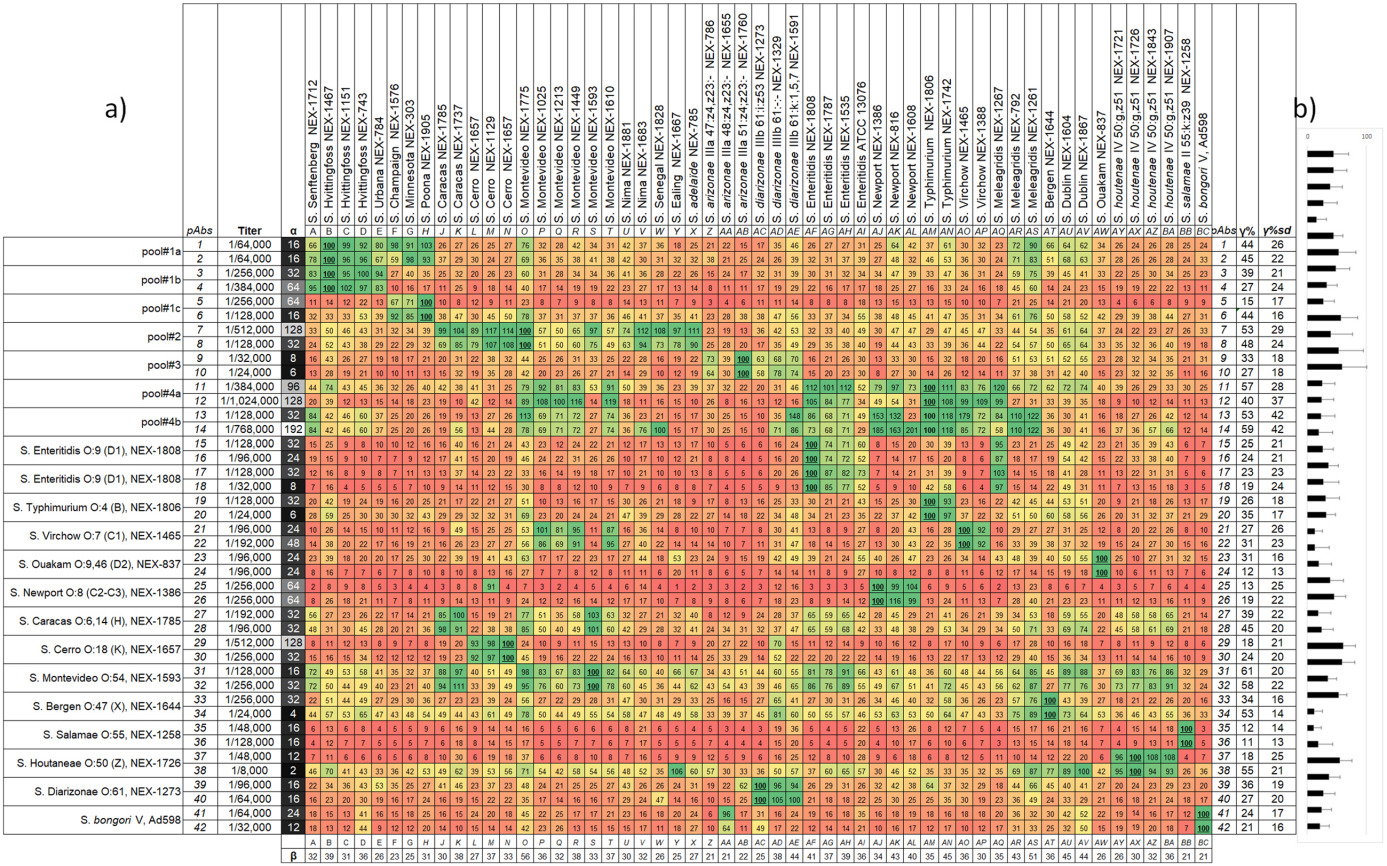
Specificity heatmap of anti *Salmonella* sera **(**A larger version of this figure is available in Supporting Information S4). The normalized colorimetric responses (*NRs%*) of 42 sera (rows 1–42) against the 54 *Salmonella* strains (columns A-BC) are shown (**a**). The second column reports the dilutions emerged from titer assessment, while the third one those used for the specificity assessment (the *α* values), which are colour coded from black (low *α* values) to white (high *α* values). At the interceptions between the serum and its immunogen, the colorimetric response is normally the maximal ones (*MR*), and is set as 100 (*NR%*, e.g. see the interceptions 41-BC and 42-BC for the two sera directed against S. *bongori* V, Ad598), while all the other *NRs%* range between 1 and 100. The *NRs%* are colour-coded from red (towards 0) to yellow (50), to green (100). The bottom row reports the *β%* values for every strain, while the two rightmost columns show the ***γ% ± γ%***_***SD***_ for every serum (see “Sera ranking by descriptors”). Graphical representation of ***γ% ± γ%***_***SD***_ for every serum (**b**).

Figure 1 reports the key properties of the 42 sera, such as immunogen, titer, and the specificity profiles against the *Salmonella* strains, as assessed by indirect colorimetric ELISA (see Supporting Information S3 for technical reproducibility considerations and signal background). Very high titres (≥ 1/192,000) resulted from the immunizations involving strains belonging to the *pool#1b-c,* *pool#2* and *pool#4a-b*, while relatively low titers were associated to the use of strains of the *pool#3* (see Supporting Information S1 for pools composition).

We then assessed the sera specificity by indirect ELISA yielding colorimetric responses (*CRs,* see Supporting Information S3 for technical reproducibility considerations), because this format does not require the structural modification (labelling) of the antibodies under examination, thus minimizing the perturbation of the binding equilibria^29^.

We thus verified the dilutions necessary to obtain similar values of the maximal *CRs* (*MRs*) when a given sera is interfaced with its immunogen (around 1.5–2.5 OD units for individual strains, and up to 2–3.5, against S*almonella* pools). We then expressed them by the arbitrary descriptor *α* (1/dilution X10E03), which normally represents a dilution 3 to 4 times lower compared to the one associated with the titer determination. During specificity determinations against *Salmonella*, the maximal colorimetric responses were around 1.5–2.5 OD units (individual strains) and up to 2–3.5 (*Salmonella* pools). We used the 42 sera at their *α* dilutions to generate a dataset (one technical replicate) in a matrix-like fashion (Fig. 1), where the rows represents the different sera (coded from *1* to *42*) and the columns the *Salmonella* strains (coded from A to BC). The results of the background signal assessment of the secondary antibody (*CRs* of 0.05–0.1 see Supporting Information S3, Sect. 1), and of the “primary antibodies” (the dilution-dependent *CRs* of 42 sera against an irrelevant proteins, resulting 0.13 ± 0.07, see Supporting Information S3, Sect. 2), indicate that the signal –to –noise ratios of the cells in Fig. 1 range from 1 to 25.

Within every row, we used the normalization with base 100 of the *CRs* (*NR%*) according to Eq. (1):

1 $$NR\% = \frac{{\text{CR of a given well}}}{{\text{CR of the well coated with the immunogen}}} \times { }100$$

which imposes *NR%* = 100 for the strain used as immunogen (e.g. intersections 5-H and 42-BC in Fig. 1), and values ranging from 1 to 100 in all of the other combinations. Technical replication of the specificity profiles shows a mean for the coefficients of variation percent (CV%) of 7% ± 5%, with few instances showing CV% up to 20% (Supporting Information S3, Sect. 4). In few instances, due to the technical reproducibility, and the molecular similarity in terms of lipopolysaccharides (LPS) and membrane proteins between some strains, the *MR* localize at the intersection with strains that were not used as the immunogen, thus yielding *NRs%* slightly exceeding 100 (see for example the intersections 7-M, and 14-AS in Fig. 1).

Each row in Fig. 1 is therefore colour coded as a “heatmap” of the *NRs%*, allowing for a visual comparison between sera specificity for the *Salmonella* strains. The heterogeneity of the *NR% values* in Fig. 1 reflects the specificity profiles emerging from the use of immunogens with difference at both qualitative (strain used) and quantitative (single strain or pool) level, and the biological diversity of rabbit immune repertoires. To highlight differences in the *NR%* dataset (54 responses per serum), one can run ANOVA pairwise comparisons either considering the individual sera as independent entities (42 sera stemming from 42 rabbits, scenario “A”, Supporting Information S4-a,c), or by accounting for the biological replicates (2 rabbits for each one of the 21 immunogens, scenario “B”, Supporting Information S4-b,d).

### Sera ranking by descriptors

As a way to rank the best sera for the subsequent development of IAs, we used multiple selection criteria that account for the results emerging from the specificity evaluations.

**Figure 2:**
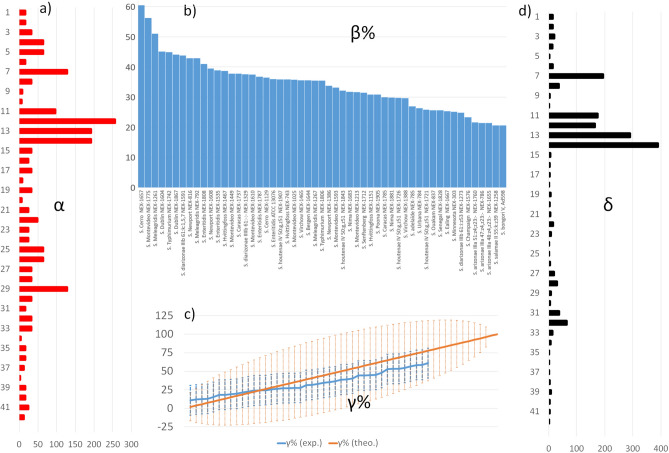
Summary of the key properties of sera descriptors. (**a**) Distribution of the *α* values of the 42 sera. (**b**) Distribution of the *β%* values of the 54 *Salmonella* strains, ranged from the highest to the lowest value. (**c**) Theoretical (orange) and experimental (blue) *γ%* ± *γ%*_*SD*_ distribution values for the “hypothetical” and simplified specificity profiles reported in Supporting S4 (54 entries) and from the real specificity assessment reported in Fig. 1 (42 entries, plotted from the lowest to the highest value), respectively. (**d**) Distribution of the *δ* values of the 42 sera.

At first, we used the *α* values to discriminate between sera that were used at either low or high dilutions to induce similar *MRs* in the heatmap (around 1.5–2.5 OD units for individual strains and up to 2–3.5 against *Salmonella* pools). The *α* values are reported in Fig. 2a, ranging between 2 (serum *38*) and 192 (serum *14*). Such 96-fold difference in the dilution to attain similar MRs can be explained by five factors, namely: (1) qualitative and quantitative differences of epitopes in the *Salmonella* strains used as immunogens; (2) rabbit’s immune system diversity; (3) different number of individual antibodies binding the *Salmonella* strain^31^; (4) different ranges of antibodies affinity; (5) a combination of all of these factors. Factor (1) accounts for the fact that immunisations against different species (*S. bongori* or *S. enterica*), subspecies (*enterica*, *salamae*, *arizonae*, *diarizonae*, and houtenae), and serovars (either isolated or combined in pools) expose rabbit’s immune response to antigens that differ for structure (proteins and O-lipopolysaccharides)^30^, localisation and immunodominance (e.g. outer membrane protein OmpA of *Salmonella enterica* serovar Typhimurium)^31,32^. While the α values resulting from the use of the 21 immunogens do not differ statistically after ANOVA and pairwise comparisons (Tukey, p < 0.05, Supporting Information S4-e), Factor (1) seems to dictate the range of the α values, and thus it is the more likely player for the observed 96-fold difference.

Factor (2) contributes to a lesser extent, because in the 21 sets of biological replicates, the CV% values are 0 for 6 instances (sera with same α, such as *1*, *2*), in the range 20–50% for 7 instances, and up to 85–105% for 8 instances (e.g. sera *13* and *14* with a eightfold difference in the α, see Supporting Information S4-e). It should be pointed out that analysis of multiple inbred mouse strains identified several (117) antigens recognized by systemic antibody responses in murine Salmonellosis^33^. Consequently, the induction of a broad immune response (Factor 1) and biological stochasticity (Factor 2) combine together to determine a multitude of antibodies (Factor 3), which differ for their variable region sequences, thus specificity and sensitivity towards individual *Salmonella* strains (Factor 4).

Another important arbitrary descriptor emerging from the specificity assessment is *β%*, which is the arithmetic mean of the *NRs%* of every sera against one strain, (mean across every column in Fig. 1). Evidently, the *β%* values depends strongly on the selection and design of the immunogens, because the more the immunogens containing strains with similar antigenic properties, the greater the *β%* values for those strains. As an example, there are eight sera originating from immunogens containing *S.* Enteritidis*,* six against *S.* Typhimurium*, S.* Newport and *S.* Virchow, and four against *S.* Meleagridis, *S.* Caracas*, S.* Cerro and *S.* Montevideo, and thus there are multiple entries in Fig. 1 attributing high *NR%* for strains belonging to these subspecies. Therefore, the descending ranking of strains according to their *β%* values (Fig. 2b) starts with many strains belonging to *pools1-a-b-c*, *pool#2* and *pools#4a-b*, while it ends with the subspecies *S.* Champaign and *S. bongori*, or those belonging to *pool#3 S. arizonae, S. diarizonae*. This reflects both a lack of a specific immunization against some strains (e.g. *S. salamae* and *S.* Adelaide) and the presence/absence of shared antigenic properties (e.g. *S. bongori*).

The *β%* values allow for a rapid identification of the strains that generate weak *NRs%*, thus highlighting those needing specificity engineering to ameliorate the immunoreaction of the sera.

We then introduced the arbitrary descriptor *γ%*, which is the average of the *NRs%* of a sera used at the **α** dilution against the 54 individual strains, and its standard deviation (*γ%*_*SD*_).

This descriptor is thus “orthogonal” to *β%* and can be related to the intrinsic specificity properties of the sera and therefore their capacity to recognize one, few, or multiple strains in a defined range of *NRs%*. *γ%* ± *γ%*_*SD*_ is therefore a measure of sera “inclusivity” against the set of *Salmonella* strains (the greater the *γ%* ± *γ%*_*SD*,_ the better the inclusivity), and “exclusivity” against the set of *E. coli* and the other bacteria species (the smaller the *γ%* ± *γ%*_*SD*,_ the better the exclusivity).

To provide a visual insight on which are the “theoretical limits” of *γ%* ± *γ%*_*SD*_ values as a function of the possible distributions of *NRs%*, let’s consider a hypothetical and simplified dataset with 54 sera, each one yielding *NRs%* of either “0” or “100” for every one of the 54 *Salmonella* strains (Supporting Information S4b).

In that theoretical scenario, the distribution of this descriptor (Fig. 2b) will range from 2 ± 14 (the least inclusive serum) to 50 ± 50 (a serum showing 27 times “100” and 27 times “0” *NRs%*, respectively), to 100 ± 0 (the most inclusive sera).

For our current set of data, the *γ%* value of every serum is reported in Fig. 1 (rightmost columns) ranging from 11 ± 13 (serum *36*) to 61 ± 20 (serum *31*), and the distribution of the values of such descriptor is confined within the ranges imposed by the theoretical distribution (Fig. 2c). From this dataset, *γ%* values were used to rank sera as single-strain specific (those in Fig. 1 with 11 < *γ%* > 30), multiple-strain specific (30 < *γ%* > 50) and those with high inclusivity (*γ%* > 50). We rapidly recognized that a ranking based only on *γ%* values can consider good sera also those having poor *α* values (e.g. serum *34* having *γ%* 53 ± 14 and *α* of 4) compared to the most performing ones (e.g. serum *7* having *γ%* 53 ± 29 and *α* of 128). We finally introduced the arbitrary descriptor *δ*, which is the result of an equation that links *α* (sera dilution) and *γ%* (sera specificity) values, in a way to promote sera with high inclusivity and *α* values. To select such equation, one has to consider that α has a broader numerical range (2–192) than γ% (12–59), and limited “steps” not equally spaced (we found only ten α values, such as 2, 4, 8, 16, 24, 32, 48, 64, 128 and 192). Therefore, the simplest way of computing δ (e.g. the linear product α and γ%, such as δ = α × γ% × C, where C is a constant) would give too importance to the titer values, and provide same δ for sera showing “symmetry” between *α* and γ% values (e.g. sera *5* and *31*, see Supporting Information S5-a). By increasing the weight of γ% in the equation by using its squared value (δ = α × γ%^2^ × C), the sera showing better combinations of α and γ% are promoted in the ranking (Supporting Information S5-b), an effect that becomes even more pronounced by using the cube of γ% (δ = α × γ%^3^ × C, Supporting Information S5-c). For these reasons, even though all of the three equations were able to rank as first the sera showing the greater inclusivity (*14, 7*, *11*, *12*) the one based on the cube of γ% was selected to generate the δ values. One can see in Fig. 2d that the *δ* values show the same profile as the *α* ones (Fig. 2a), but with “enhanced” or “smoothened” entries. Indeed, sera that were good for their *α* values but not “inclusive” (e.g. *4*, *5*, *29*) or good for their inclusivity but with scarce *α* (e.g. *34*, *38*) are “filtered” off by such equation, while sera with the best inclusivity and *α* (e.g. *7*, *11*, *12*, *13*, *14*, see Fig. 2d) are “enhanced”.

Ranking by *δ* promotes the selection of sera that target among the most important *Salmonella* serovars, such as those belonging to the *pool#2* and *pool#4a-b*, (see Supporting Information S1), and that can be used at high dilutions, which is beneficial for both technological and sera optimization needs.

By accounting for descriptors values, we selected sera *12* and *14* for a more exhaustive specificity screening against the *Salmonella* strains (inclusivity assay) and the *E. coli* strains and the other bacteria listed in Supporting Information S1 (exclusivity assay). For the sake of specificity comparisons and “benchmarking”, we selected a commercial antibody targeting a common *Salmonella* antigen (goat pAb by the Kirkegaard & Perry Laboratories, *KPL*), and the colorimetric responses against a strain well recognized by the majority of the anti-*Salmonella* sera (*S.* Virchow), instead of *MR*, were used to yield the *NRs%*. The *KPL* pAb is prepared via a proprietary purification procedure that yields good inclusivity and excellent exclusivity, according to the manufacturer, and is thus an excellent benchmark for our strategy regardless of any subsequent purification step.

**Figure 3:**
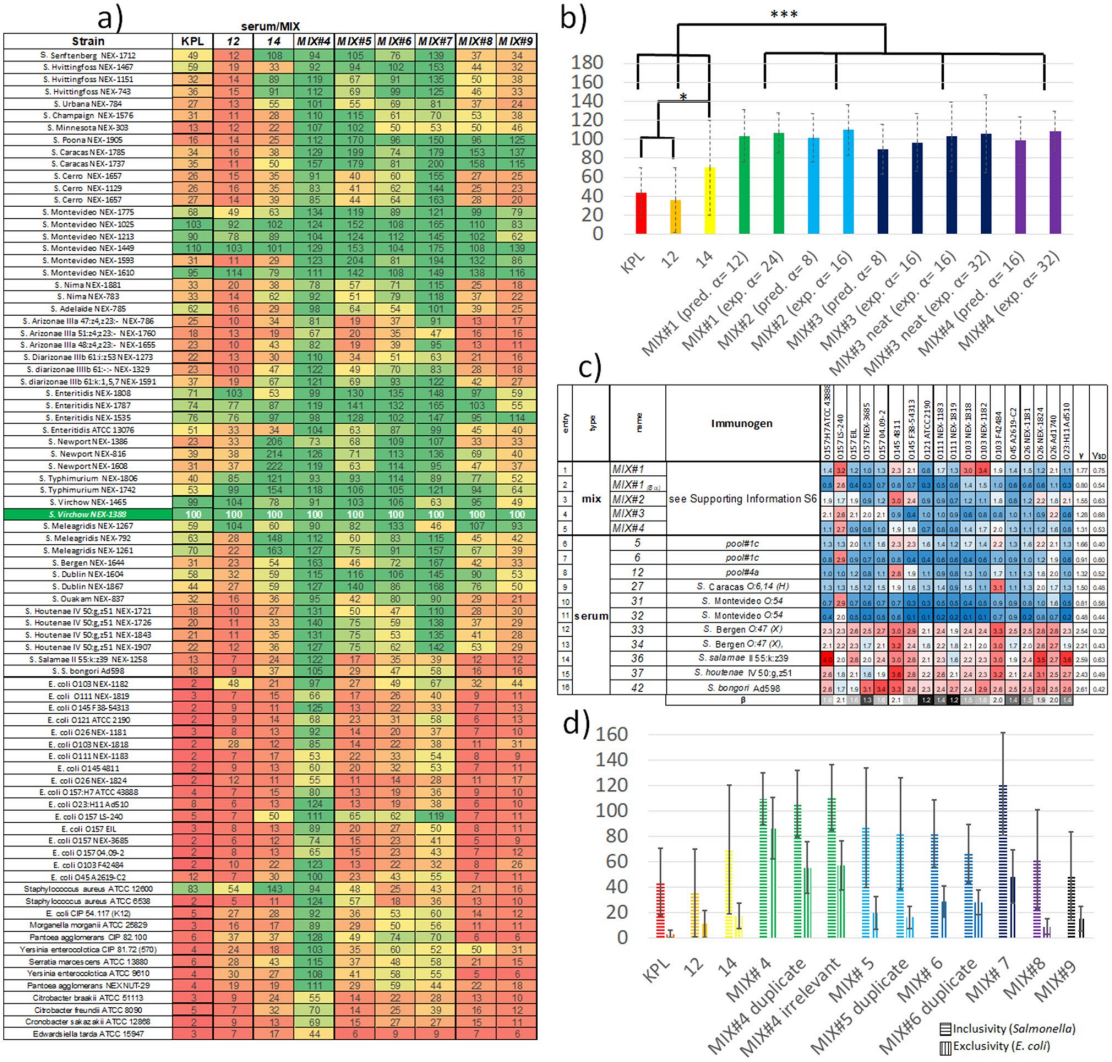
(**a**) Specificity heatmap of sera and *MIXs*, colour coded from red (towards 0 of the *NRs%*), yellow (50), and green (100). (**b**) γ% ± γ%_SD_ values of the *MIXs#1–4* along with their predicted values (pred.), and of the reference sera *12, 14 and KPL*. *MIX#* means a mixture realised by using the already diluted sera, while *MIX# neat* refers to mixtures realized starting from the corresponding neat sera. Inside the parenthesis, the α values of predicted or experimental mixtures are reported. (**c**) *E. coli* exclusivity heatmap of the MIXs and some individual sera, reporting the colorimetric values (no data normalization) and colour coded from red (higher cross-reactivity towards *E. coli*), white, and blue (lower cross-reactivity towards *E. coli*). Colorimetric values of 1.33 correspond to about 35–40% of the colorimetric responses in the *Salmonella* inclusivity heatmaps, in Fig. 1 and here in Fig. 3a). (**d**) Average inclusivity (γ% ± γ%_SD_ values for *Salmonella*) and exclusivity γ% ± γ%_SD_ values for *E. coli*) of the best sera and *MIXs*. *p < 0.05, ***p < 0.001.

The comparison of these three inclusivity/exclusivity heatmaps (first three columns in Fig. 3a) shows similar distribution of the *NRs%*, such as more than 50 for some *S.* Montevideo*, S.* Enteritidis*, S.* Typhimurium and *S.* Meleagridis subspecies (which are among the most important serovars to detect), and lower than 50 for the majority of the other strains. This result in *γ%* ± *γ%*_*SD*_ values of 36 ± 34 for *12*, 70 ± 50 for *14* and 44 ± 26 for the *KPL* pAb, which means a greater inclusivity of *14* compared to *12* and *KPL* for the selected set of strains (p < 0.01, Fig. 3b). As expected, the *KPL* pAb shows the best exclusivity (*γ%* ± *γ%*_*SD*_ = 6 ± 14), followed by serum *12* (13 ± 11) and serum *14* **(**22 ± 21), the latter being significantly less exclusive than the *KPL* pAb (p < 0.01).

### Specificity harmonization of serum mixtures

Taken together, these observations prompted us to find a rational and potentially general solution to select sera with great **α** to generate *MIXs* showing high *NRs%* towards the inclusivity set of strains *(Salmonella*), and low *NRs%* towards the strains belonging to the exclusivity set (*E. coli* and the others).

From the combinatorial point of view, the possibilities for creating *MIXs* by selecting a certain number (n) of sera among the 42 available are 861 (n = 2), around 112,000 (n = 4) and about 120 million (n = 8), thus rapidly reaching too large experimental spaces to be screened. Even by reducing the library size to 32 sera (the 4 with maximal δ and the 28 against individual strains), or down to 21 (by eliminating the worst biological replicates), there are still hundreds to thousands of MIXs to be prepared and screened. Furthermore, one wants to use the least possible number of sera, firstly because a single polyclonal response (serum) has already a large number of antibodies in it, and secondly because the simpler the mixture, the higher the chances to reproduce the results and the easier the characterizations by the current protocols of recombinant antibodies production involving mass spectrometry^34^.

We thus designed four *MIXs* by taking advantage of sera ranking by *δ*, which suggest the use of 7, *11*, *12, 13* and *14* as “bases” for specificity engineering, and by using simple algorithms in an electronic spreadsheet to identify the sera to be used to increase the *NRs%* against given strains. It is indeed sufficient to determine the mean *NRs%* for a subset of sera against a given strain, to generate “artificial heatmaps” representing the predicted specificity profiles of a given *MIX* (Supporting Information S6b). This is equivalent to compute the *β%* values for a subset of the sera library. Once the best sera are identified, they are introduced in a *MIX* at the 2*α* dilution (a further dilution to avoid colorimetric response saturation due to the contemporaneous presence of antibodies belonging to different sera targeting the same epitopes, and by the volumetric ratios determined by Eq. (2).

2 $$Volumetric \,ratio \left( n \right) = { }\frac{\alpha max}{{\alpha \left( n \right)}}$$

As an example, a mixture of three sera with *α* = 128 (serum *12*), 64 (serum *5*) and 32 (serum *30*) will mean the use of 1 volume of *12* (diluted 1/256,000), 2 volumes of *5* (diluted 1/128,000), and 4 volumes of *30* (diluted 1/64,000).

By this way, *MIX#1* and *MIX#2* with 11 sera, *MIX#3* with 7 sera, and *MIX#4* with 8 sera, were prepared (see compositions and immunogens in Supporting Information S6). More into the detail, *MIX#1* and *MIX#4* are prepared with sera whose immunogens contain strains representing around 20% of the overall *Salmonella* serovars considered in this study (potential strain coverage), while *MIX#2* and *MIX#3* have twice that value (40%).

The *γ%* ± *γ%*_*SD*_ values of the four *MIXs* (evaluated as single technical replicates) are 107 ± 21 (*MIX#1*), 111 ± 27 (*MIX#2*), 97 ± 30 (*MIX#3*), and 110 ± 20 (*MIX#4*), result significantly higher than the best individual sera, (*12*, *14*, and *KPL*, p < 0.001) but do not differ from each other statistically (Fig. 3b and Table S6a in Supporting Information S6) in spite of the different potential strain coverage (20% or 40%). Values exceeding 100 originate from the fact that the NRs% do no longer colocalize with the MRs, but are obtained by the normalization against the commonly well recognized strain S. Wirchow, O:7 (C1) NEX-1388.

The inclusivity heatmaps of these *MIXs* (Supporting Information S6, Figure S6b) clearly show harmonization of the *NR%* for all of the 54 *Salmonella* strains, except for *MIX#3,* which was expressively designed to harmonize the *NRs%* only against the serovars represented in the *pools#1–4,* which all belong to the species *enterica* (an in particular the subspecies *enterica*, *arizonae* and *diarizonae*) thus showing reduced *NRs%* improvements for the subspecies *houtenae*, *salamae* and the species *S. bongori*.

There is a good agreement between the experimental and predicted *NR%* values, resulting in artificial and experimental datasets that does not show statistically-significant differences between them (see Fig. 3b and Supporting Information S6, Figure S6b and Table S6b), and that highlight which strains benefit from *MIX* design (e.g. see the comparison between serum *14* and *MIX#4* in Fig. 3a). Interestingly, the volumetric ratios for *MIX* generation were effective also at the preparative scale, meaning the blending of neat sera in a MIX that is then diluted at the α value, instead of combining the 10X diluted sera (used for titer and specificity assessment) at the final dilution.

Indeed, the comparison of *MIX#3* produced from diluted constituents or from *neat sera* (*MIX#3*_*(exp. α*=*8)*_ and *MIX#3 neat*_*(exp. α*=*16)*_*, *Fig. 3b) shows that the overall inclusivity is undistinguishable, even by a further dilution (*MIX#3 neat*_*(exp. α*=*32)*_ see Table S6b, Supporting Information S6).

Taken together, the results highlight the feasibility of using a simple “macroscopic” output (the colorimetric response, function of the antibody density in every well) as a way to rank sera that, upon rational mixing, were suitable to engineer MIX specificity and achieve a 50%-100% increase of *γ%* compared to the most inclusive sera (*12* and *14*, see Fig. 3b).

For the exclusivity assessment of this set of four *MIX*, it should be pointed out that there could be some “physiologic” cross reactivity towards *E. coli* serotypes depending on the immunogen composition, namely the presence *Salmonella* strains that share antigenicity with an *E. coli* strain (e.g. *S. bongori* subspecies)^35^.

The exclusivity heatmap in Fig. 3c show the colorimetric responses (from 0.066 to 4) reflecting different levels of cross-reactivity of the 4 *MIXs* against 18 *E. coli* strains. In this case, there is no need of colorimetric response normalization against a specific strain, because there is no E. coli strain that act both as analyte and immunogen (as previously seen in the *Salmonella* inclusivity assay). The exclusivity ***γ ± γ***_*SD*_ values of the four *MIX* are 1.77 ± 0.75 (*MIX#1*), 1.55 ± 0.63 (*MIX#2*), 1.28 ± 0.68 (*MIX#3*), and 1.31 ± 0.53 (*MIX#4*) with no differences from the statistical point of view (Supporting Information S7, Table S7a).

Upon *MIXs* further dilution (8α) *γ* values obviously decrease (p < 0.05, see entries 1–2 in Fig. 3c and Supporting Information S7), suggesting the possibility of diluting one or more constituent of a given MIX (*α* value optimization) as a way to control cross-reactivity.

Since *MIX#4* seems the best candidate for further optimizations in view of its *γ%* values (highest inclusivity), narrow *γ%*_*SD*_ (harmonised colorimetric response), and number of constituents (8 sera), we determined the exclusivity of its constituent sera and of closely related ones (see entries 6–16 in Fig. 3c and Supporting Information S7). It turned out that the sera *33*, *34*, *36*, and *42* heavily cross-react with most of the 18 *E. coli*, while *5*, *12*, and *27* showed better exclusivity profiles (p < 0.05, Supporting Information S7). It is not surprising that the serum elicited by *S. bongori* heavily cross-react with the library of *E. coli* strains, being these microorganisms closely related^36^. Molecular similarity may be the explanation why both biological replicates against *S.* Bergen (sera *33* and *34*) and the ones against *S. salamae* (*36*) and *S. houtenae* (*37*) show such cross-reactive profiles.

The colorimetric results from both the inclusivity and exclusivity assessments account for the presence of a multitude of antibodies belonging to different sera, which may participate in both “homologous” (from the same serum) or “heterologous” (from different sera) fashion for avidity against the antigens, which are in some instances shared with *E. coli* strains^35,37^.

The interested reader may found here^38^ a recent summarization on the complexity of polyclonal sera avidity assessment, which has been developed especially in the fields of vaccine research to try finding correlations between thermodynamic parameters of binding and the resulting neutralizing effects. It is out of our scope to get such thermodynamic characterizations and molecular insights, but rather to use a multitude of antibodies belonging to different sera, and an incubation conditions (1 h at room temperature) gentle enough to allow reaching binding equilibria with the *Salmonella* antigens exposed onto the ELISA wells. In these conditions, a simple macroscopic reporter (colorimetry) is an index of the antibody density in every well containing a *Salmonella* serovars, because similar colorimetric responses imply similar numbers of antibodies immunosorbed. Sera selection and dilutions are the variables modulating the chemical spaces for the occurrence of such equilibria, towards the theoretical limit of 100 ± 0 *γ%* ± *γ%*_*SD*_ (Figure S4b in Supporting Information S4).

### Serum mixtures optimization

Some considerations prompted us to investigate whether we could further optimize *MIXs* composition by reducing the number of sera (3–5), and tailoring their dilutions. We produced *MIX#5* with 3 sera, (*5*, *12*, and *27*), *MIX#6* with 5 sera (*15*, *17*, *19*, *22*, and *26*), and *MIX#7* with 4 sera (*27*, *29*, *32* and *33*). See Supporting Information S6 for their composition.

Their specificity evaluation (Fig. 3a,d) clearly reports that *MIXs#5-6-7* display harmonized specificity profiles, with *MIXs#7* showing γ% ± γ%_SD_ greater than those of the best sera (p < 0.001), while *MIXs#5 and MIXs#6* performing better than *12* and KPL (p < 0.001), as revealed by pairwise comparisons (see Tables in Supporting Information S6). Upon technical replication, one can see that the inclusivity datasets of the MIXs are undistinguishable, even upon addition of irrelevant sera as a way to “foster” possible background increases due to the number of constituents in the *MIXs* (p < 0.05 for *MIX#4*, see Table S6c in Supporting Information S6). Regarding the exclusivity, *MIX#5* and *MIX#6* show better exclusivity profiles compared to *MIX#4*, even though technical replication of *MIX#4* by mixtures having or lacking the additional irrelevant sera resulted in better exclusivity profiles compared to the initial attempt (see Tables in Supporting Information S7).

Even though *MIX#7* shows a huge improvement in terms of inclusivity compared to the other *MIXs* and sera, *MIX#5* represents the best compromise between specificity improvement and number of constituents to be further optimized (only 3, compared to 11 constituents for *MIX#4* and 4 for *MIX#7*).

**Figure 4:**
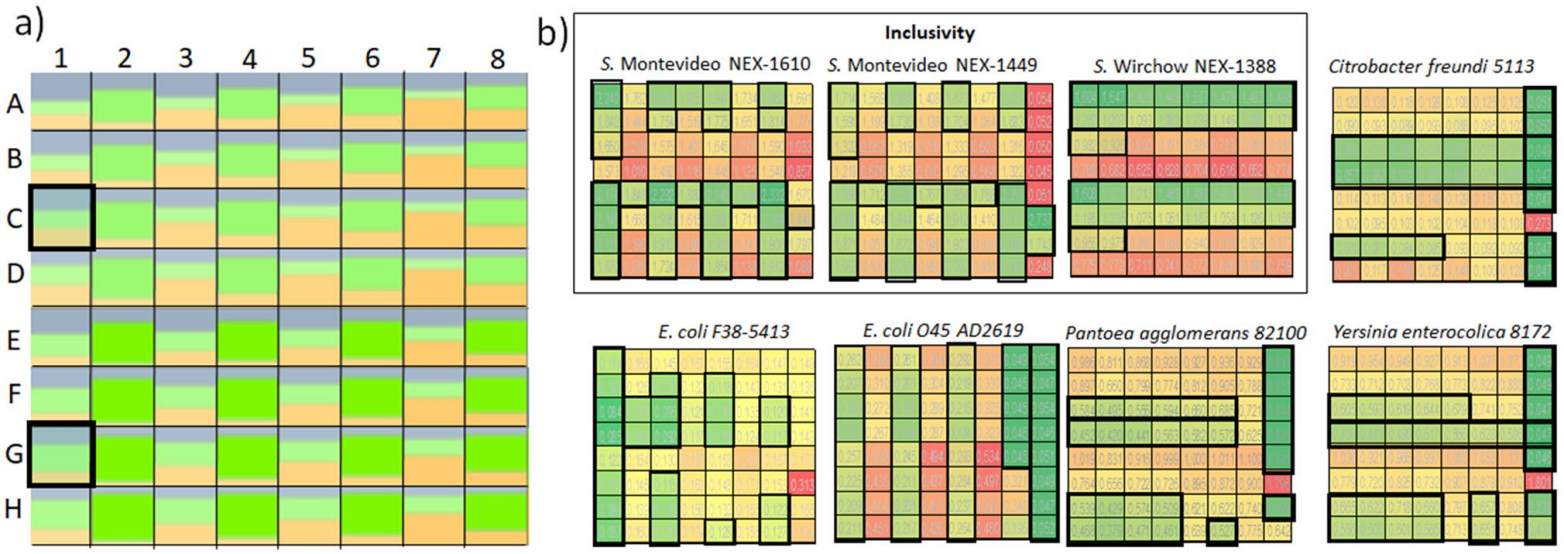
Results of the MIX optimization (*α* values screening). (**a**) Color-coded dilutions of the three sera constituting the optimal *MIX* (*12* = grey, *27* = green, *5* = yellow), where in each combination (A1–H8) the smaller the individual colour region, the higher the dilution of the corresponding constituent. As an example, the combination in E3 has *12*, *27* and *5* equally diluted at 1/128,000, while the combination in H8 display *12* 1/384,000, **27** 1/32,000, and *5* 1/64,000. For a list of all of the dilutions screened and their approximate concentration values, see Supporting Information S8. (**b**) Heatmaps of the colorimetric responses of the 64 *MIXs* against plates coated with *Salmonella* strains (inside the rectangle labelled as Inclusivity) or the other negative controls for the exclusivity assessment. The best colorimetric responses (high towards *Salmonella*, low towards the other bacteria) are colour coded from yellow to green, and are surrounded by black squares/rectangles, which facilitates the visual identification of the common conditions that ensure both inclusivity and exclusivity maximization at a same time (C1 and G1 in **a**).

These results prompted us to select *MIX#5* as the candidate for the optimization of the dilutions of its individual constituents (**α** value optimization). To this aim, we planned and executed a complete factorial design with the factors being the three individual constituents of *MIX#5* (sera *5*, *12*, and *27*) and the levels being four dilutions for each constituent in the range 1/32,000 (around 200 ng/mL IgGs) to 1/384,000 (around 15 ng/mL IgGs, see Supporting Information S8). The experimental plan implies the generation of 4^3^ = 64 *MIXs* (Fig. 4a) showing different volumetric ratios between the three sera, and their screening against eight selected strains.

Three *Salmonella* strains were selected because they were recognized by all of the sera and *MIXs* with similar extents (e.g. *S.* Montevideo *NEX-1610, S.* Montevideo *NEX-1449, S.* Virchow *NEX-1388*), while 5 strains were selected because they were source of cross-reactivity (*E. coli F38-5413, E. coli O45, Pantoea agglomerans, Yersinia enterocolica, Citrobacter freundi*). The resulting dataset of 64X8 colorimetric responses was visually inspected (Fig. 4b) and subjected to software-based optimisation (sDoE Supporting Information S8), by having as goal the individuation of the optimal combinations of inclusivity and exclusivity. By visual inspection of the heatmaps in Fig. 4b, there are many combinations fulfilling one of these two requirements, such as greatest inclusivity (those with high volumetric ratios of serum *12*), greatest exclusivity against *E. coli* (those with low volumetric ratios of *27*) or greatest exclusivity against the other genus (those with low volumetric ratios of *5*).

There are only two combinations that maximize the specificity requirements, both of them employing *12* at *α* = 256 and *5* at *α = *192, but slightly differing for *27* dilutions, (*α* = 256 or 128 for the combinations at the intersection **C1** and **G1** in Fig. 4a, respectively). Notably, also the sDoE approach identified the combination **G1** as the best compromise (Supporting Information S8).

We thus identified the combination C1 as *MIX#8* and G1 as *MIX#9*, and compared their properties with the other sera and *MIXs*, after the usual inclusivity/exclusivity assessment (Fig. 3).

Both *MIXs* display exclusivity in the same range than the commercial reference *KPL* and serum *12* (p < 0.001), but *MIX#8* has the same inclusivity as the parent mixture *MIX#5*, while *MIX#9* display lesser performances (lower inclusivity, p < 0.001). Therefore, these two *MIXs* show an improvement of the *α* values of their “limiting reagent” (serum *27*), which can be used at a much greater dilution (4X – 8X more) compared to *MIX#5*.

Based on all of these observations, the possibility of exploiting this sera development strategy to tune the specificity in a customized fashion is demonstrated (Fig. 3). If *Salmonella* detection has to be maximized whilst possible cross-relativities towards *E. coli* are less concerning, *MIX#7,* *MIX#6* and *MIX#5* would be the best solutions (Fig. 3d). If a good compromise between inclusivity for *Salmonella* and exclusivity for the other strains, *MIX#8* and *MIX#9* would be the best choice (Fig. 3d).

## Conclusions

We have presented a rational strategy to develop mixtures of antibodies able to maximize and harmonize the specificity profiles against 54 targets (in this case *Salmonella* strains) that share different degrees of similarity among them and between closely related analytes (*E. coli* strains).

This strategy is based on the production of a sufficient number of sera to cover the different features of the analytes (antigenic properties), and on the evaluation of their relative specificity profiles emerging from indirect ELISA assays. Rational parametrization of the resulting datasets allows predicting the responses of mixtures of different sera towards the selected analytes, where few sera resulting from immunisation against pools of *Salmonella* strains, thus showing broad specificity, can serve as a “base” for a further specificity tuning, thanks to the addition of sera with narrower specificity profiles (e.g. those involving immunisation against individual strains). This process allows the *design* of mixtures with custom specificity profiles rather than their mere *identification* by large combinatorial screenings. In our case, it meant the design and screening of only a dozen of serum mixtures to maximize and harmonise specificity with the least possible constituents, thus requiring feasible characterisations (10^3^–10^4^ ELISA wells), instead of screening among the multitude of possible combinations (10^6^–10^7^ ELISA wells).

This strategy thus avoids waste of biological resources (animals to produce sera), chemical resources (reagents for the screening of the immunoassay properties), human resources (for data production and interpretation), and time. Most importantly, it represents a general strategy to generate custom specificity profiles by addition of easily available constituents, rather than the use of more elaborated and case-specific protocols (such as chromatographic purifications). The limits of this strategy rely mostly on the technical reproducibility of the ELISA system available, because in the generation of the descriptors, the precision on both titer and *NR%* determinations (see Supporting Information S5, Sect. 2) affect the possibility of ranking by statistically significant differences.

In the specific *Salmonella* case, there are different possibilities to exploit the optimized mixtures, such as in competitive- or sandwich-based immunoassays. In the former case, bacteria pools can be immobilized on suitable surfaces (coating/coupling on ELISA wells or onto fluorescent µbeads), while in the latter the IgG fraction needs to be noncovalently (physisorption, protein A) or covalently (carbodiimmide chemistry/ periodate oxidation) bound to those surfaces. Every aforementioned combination has its own “pros” and “cons”, but the combinations that does not imply (competitive indirect) or limit (sandwich indirect) pAbs chemical modification are the most likely to “translate” the specificity profiles obtained by our optimization strategy. In general, this strategy is very suitable for the development of bioanalytical assays where a multitude of analytes displaying molecular similarity need to be discriminated, thus requiring a signal threshold. These are the typical requirements of ELISA- and flow cytometry-based detection of drugs belonging to different class of actions, or proteins having immunomodulatory effects such allergens and gluten fractions. This strategy generates datasets that are in between what starts to be too large to be analysed by mere man-made operations in a spreadsheet software, and what is too little for the mathematical models and software necessary for big-data handling (e.g. next-generation sequencing).

Nonetheless, for this size of datasets, there are several mathematical and statistical models that can be employed to optimize and find trade-offs between the conflicting objectives of inclusivity and exclusivity maximization of a given mixture, such as multi-objective optimization in closed loop fashion^39^, by taking advantage of very rapid problem description, computational prediction, and experimental verification.

At last, this strategy fits well with the recent advances on rabbit immune response exploitation by combined Mass-spectrometry-Next generation sequencing (NGS) protocols. Those protocols require on the one hand to obtain the genetic information behind antibody production (whole antibodies “genotyping” by NGS), and on the other hand to obtain the variable sequences (“phenotyping”) from the specific antibodies after their isolation (immunoaffinity chromatography) and proteomic determination (enzymatic digestion plus high –resolution mass spectrometry), to allow for variable sequences alignment by bioinformatics tools and recombinant antibody expression^40,41^. Due to the progressive reduction of NGS costs, and presence of very high performance mass spectrometers, specific IgG isolation from the optimized mixtures will therefore be a convenient tool to expand the (already large) repertoire of variable sequences to be paired and recombinantly expressed for the generation of “cocktails” of recombinant antibodies targeting complex analytes^19,42^.

## Methods

### Production of polyclonal antibodies (sera)

All of the experimentation involving animals was done under the frame of the ethical protocol CE/Sante/E/001 (immunization and production of sera/polyclonal antibodies) approved by the ethical committee of CER Groupe (agreement nb. LA1800104). The agreement LA1800104 was bestowed by the Federal Public Service of the Walloon Region (Belgium). The experimentation respected the legislation in force at the moment of the studies, thus following the guidelines established at the European level (Directive 2010/63/EU revising Directive 86/609/EEC on the protection of animals used for scientific purposes), Belgian level (Arrêté royal relatif à la protection des animaux d'expérience, AR 2013/05/29), and Regional level (Code Wallon du Bien-être animal 03/10/2018). Polyclonal antibodies were raised in rabbit by subcutaneous injection of 10^9^ CFUs of inactivated bacteria (isolated strains or mixtures, see list in Supporting information S1) emulsified with Freund’s complete adjuvant for the first injection or Freund’s incomplete adjuvant for all following injections (Becton Dickinson Benelux, Erembodegem, Belgium). Injections were administered on a fortnightly basis and then, from the third injection onward, at the rhythm of one injection every 28 days^43,44^. Test bleeds were collected 10 days after each immunization (from the third immunization onward).The blood was centrifuged, and the collected serum was stored at − 20 °C until used. An aliquot of such serum was diluted 1/10 in a solution of 50% assay buffer (phosphate buffer 65 mM, NaCl 150 mM, 0.2% gelatin, 0.05% Tween 20, 0.01%, 8-anilino-1-naphthalenesulfonic acid ammonium salt, and ascorbic acid 28 mM) and 50% ethyleneglycol, yielding diluted sera solutions that were kept at -20 °C prior to their use for titer, specificity assessment and MIX production.

### General procedure for the colorimetric indirect ELISA

96-well plates (Nunc Immuno F8 maxisorb, Thermo Scientific) were coated overnight at room temperature with a solution of inactivated bacteria either isolated or as bacteria pools (10^7^ CFUs overall) in a 50 mM carbonate buffer (pH 9.6). The wells were washed three times with washing buffer (0.15 M NaCl and 0.05% Tween 20 in distilled water), and blocking was performed with saturation buffer (goat plasma or rabbit serum diluted 2,000X in 50 mM carbonate buffer pH 9.6, depending on the origin of the secondary antibody) for at least 2 h at 37 °C before washing again. Wells equilibration at neutral pH was done by incubating at least 10 min in conservation buffer (phosphate buffer 20 mM, NaCl 150 mM, glycerol 2%, gelatin 0.1%, pH 7.4). The test sera were diluted in the assay buffer (phosphate buffer 65 mM, NaCl 150 mM, 0.2% gelatin, 0.05% Tween 20, 0.01%, 8-anilino-1-naphthalenesulfonic acid ammonium salt, and ascorbic acid 28 mM), and the wells incubated for 1 h at room temperature. BacTrace Anti-*Salmonella* CSA-1 Antibody (SeraCare, Kirkegaard & Perry Laboratories, reference# 5310-0322) produced in goat was reconstituted according to the instructions furnished by the supplier and used as primary antibody upon dilution 1:16,000 in assay buffer. After three washes, anti-Rabbit IgG (whole molecule)–peroxidase antibody produced in goat (Sigma, Saint-Louis, USA, reference# A6154-1ML) diluted 1:10,000 in assay buffer (for the primary rabbit sera) or a anti Goat IgG-Fc Peroxidase antibody produced in rabbit (Bethyl Laboratories reference#A50-104P) diluted 1:20,000 in assay buffer (for the primary goat pAb) were added and the plate was incubated for 30 min at room temperature. After another three washings step, the chromogenic substrate 3,3′,5,5′-tetramethylbenzidine (TMB) was added, and the wells were incubated at room temperature for 30 min. The colour-producing enzymatic reaction was stopped by adding 1.8 N H_2_SO_4_, and the absorbance was measured at 450 nm in a Skanit plate reader (Thermo Fisher Scientific), which declares linearity within 0–4 units of optical density, then nonlinearity occurs (values up to 6). During titer assessment, at very low dilutions some responses reached saturation, but were never considered (titer is determined as the dilution yielding the optical density value closer to 1). During specificity determinations against *Salmonella*, the maximal colorimetric responses were around 1.5–2.5 OD units (individual strains) or up to 2–3.5 OD units (*Salmonella* pools), while against some E.coli strains some sera showed high colorimetric responses (See Fig. 3c, with serum 36 showing four responses higher than 3 OD units and one reaching 4 OD units).

### Sera titer assessment

The general procedure for the indirect colorimetric ELISA was used, by interfacing two-fold serial dilutions of each diluted sera (variable ranges, such as from 1/500 to 1/64,000 or from 1/16,000 to 1/2,048,00, depending on the serum) against wells coated with the strain or the pool of strains used as immunogen. Two types of nonspecific binding (NSB) were evaluated: (1) the appearance of colour in coated wells in the presence of peroxidase antibody but in the absence of sera, (NSB against wells displaying the coating, NSBc), and (2) the appearance of colour in noncoated wells blocked with saturation solution 1/2000 (NSB against wells non coated, NSBnc) and interfaced with sera at the lowest dilution (e.g. 1–500 or 1/16,000, depending on the range of dilutions used during the titer). The titer is expressed as the dilution yielding the colorimetric response closer to 1 unit of optical density.

### Specificity assessment of individual sera

The general procedure for the indirect colorimetric ELISA was used, by interfacing diluted sera with different strains (see the example in Supporting Information S3), by using a final dilution generating a maximal colorimetric response (MR) close to 3.3–3.5 units of optical density. This normally corresponds to a dilution 4-8X lower than the one yielding the titer, and is reported in the text as the value of the arbitrary descriptor *α*. Only one type of nonspecific binding (NSB) was evaluated (NSBc, because NSBnc was already determined during titer assessments).

### Production of serum mixtures

Mixtures were produced according to the volumetric ratios derived from Eq. (2), by blending the sera at a 2* α* dilution (MIX) or the neat sera (MIX_(neat)_).

### Specificity assessment of serum mixtures

The general procedure for the indirect colorimetric ELISA was used, by interfacing MIX or MIX_(neat)_ with different strains (see the example in Supporting Information S3), by using a final dilution generating a maximal colorimetric response (MR) close to 3.3–3.5 units of optical density. One type of nonspecific binding (NSB) was evaluated: (1) NSBc, i.e. the appearance of colour in coated wells in the presence of the peroxidase antibodies but in the absence of MIX or MIX_(neat)_.

### α values optimization by statistical design of experiments (sDOE)

We generated a complete factorial design by encoding three factors (sera *12*, *27*, and *5*) and four levels of dilution (#1, #2, #3 and #4, see Supporting Information S8) by using the appropriate utility of Minitab 18 software. The resulting 64 combinations (mixtures of sera) were generated by loading the three sera (diluted 1/10 in a solution of assay buffer/ethyleneglycol 1:1) into the cartridges (Hewlett Packard T8 + Dispensehead Cassette) for the use in a digital dispenser (Tecan D300E), and by translating the experimental plan from the Minitab software (Minitab 18 Statistical Software, 2010. Computer software, State College, PA: Minitab, Inc. www.minitab.com) into a Microsoft Excel (2016) file, imported in the Tecan D300 Digital Dispenser Software (v3.3.2, https://lifesciences.tecan.com/products/liquid_handling_and_automation/tecan_d300e_digital_dispenser). The mixtures were thus instantaneously “printed” inside each well, which was coated according to the plan illustrated in Fig. 4. Then, the steps indicated in the general procedure for the indirect colorimetric ELISA applies. One type of nonspecific binding (NSB) was evaluated (NSBc), the appearance of colour in coated wells in the presence of the anti-rabbit peroxidise antibody, but in the absence of the MIXs. All the heatmaps in the body text and Supporting Information were prepared with Microsoft Excel (2016).

## Supplementary information

Supplementary Information.
