# Supplementary material for: A general strategy to control antibody specificity against targets showing molecular and biological similarity: Salmonella case study
Source: Sci Rep. 2020 Oct 28;10:18439. doi: 10.1038/s41598-020-75285-1 (PMC7595100; doi:10.1038/s41598-020-75285-1)
Supplement: Supplementary file 1 — Supplementary Information. [file 41598_2020_75285_MOESM1_ESM.docx]

_­­­_

**A general strategy to control antibody specificity against targets showing molecular and biological similarity: *Salmonella* case study**

**In memory of Patrice Arbault**

Marega R.^1*^, Desroche N.^2^, Huet A.-C.^1^, Paulus, M.^1^, Suarez Pantaleon C.^3^, Larose D.^2^, Arbault P.^2^, Delahaut P.^1^ and Gillard N.^1^

**^1^ CER Groupe, Analytical Laboratory, Rue du Point du Jour 8, 6900 Marloie, Belgium**

**^2^ NEXIDIA S.A.S., Rue de Mayence 15, 21000 Dijon, France**

**^3^ Unisensor S.A., Rue Louis Plescia 8, 4102 Ougrée (Liège), Belgium**

To whom correspondence should be addressed: Dr. Riccardo Marega, r.marega@cergroupe.be

**Supporting Information S1: Bacteria involved in this study and typical coating scheme for specificity assays**

**Tshe rabbitXst sertiter aasebodyitture which is beneficial for both technological and pAbs optimization needs. able S1. List of the bacteria strains** used in this study, summarizing use (only as analyte, to verify sera specificity, or also as immunogen, to elicit sera), species (*S. enterica*, *S. bongori* or *pool,* meaning a combination of several strains), subspecies (*arizonae, diarizonae, houtenae* or *enterica*), serovars, O Group, strain.

Pool preparation required equal amounts of bacteria in order to achieve an overall amount of *Salmonella* strains 1X10E07 CFU. For instance, pool#1b, #1c and #3 are made by three strains, which were mixed together at 1:1:1 volumetric ratios from stock solutions of 1X10E09 CFU. The resulting mixture was then diluted 100 times to get 1X10E07 CFU as final concentration. Pools #1a and #2 are composed of six *Salmonella* strains, which were mixed together at 1:1:1:1:1:1 volumetric ratios from stock solutions of 1X10E09 CFU. The resulting mixture was then diluted 100 times to get 1X10E07 CFU as final concentration. The only exception of these volumetric ratios applies for pool#4a, composed of four strains, where *S.* Enteritidis was used at a double concentration (2:1:1:1). The reason was a mistake during immunogen preparation, which did not pose dramatic problems *a posteriori*, being the immune response fairly dose independent over similar ranges (e.g. similar amounts of immunogens can be used to elicit antibodies in rabbits, goats and Llamas, in spite of the huge difference of their relative body mass).

**Supporting Information S2: Comparison between intravenous and intradermal routes for anti-*Salmonella* sera and their titer values.**


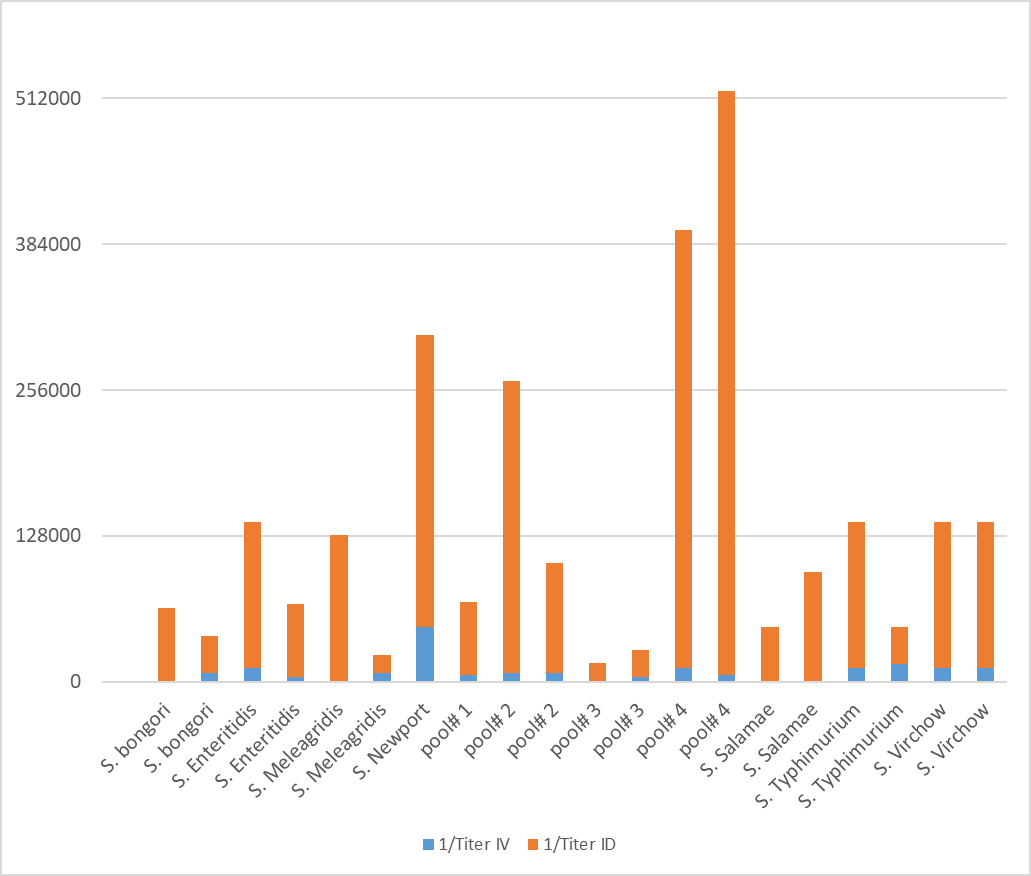


**Figure S2a** Titer evolution of after intradermal (ID) or intravenous (IV) administration of the thermally inactivated *Salmonella* strains and pools.

**
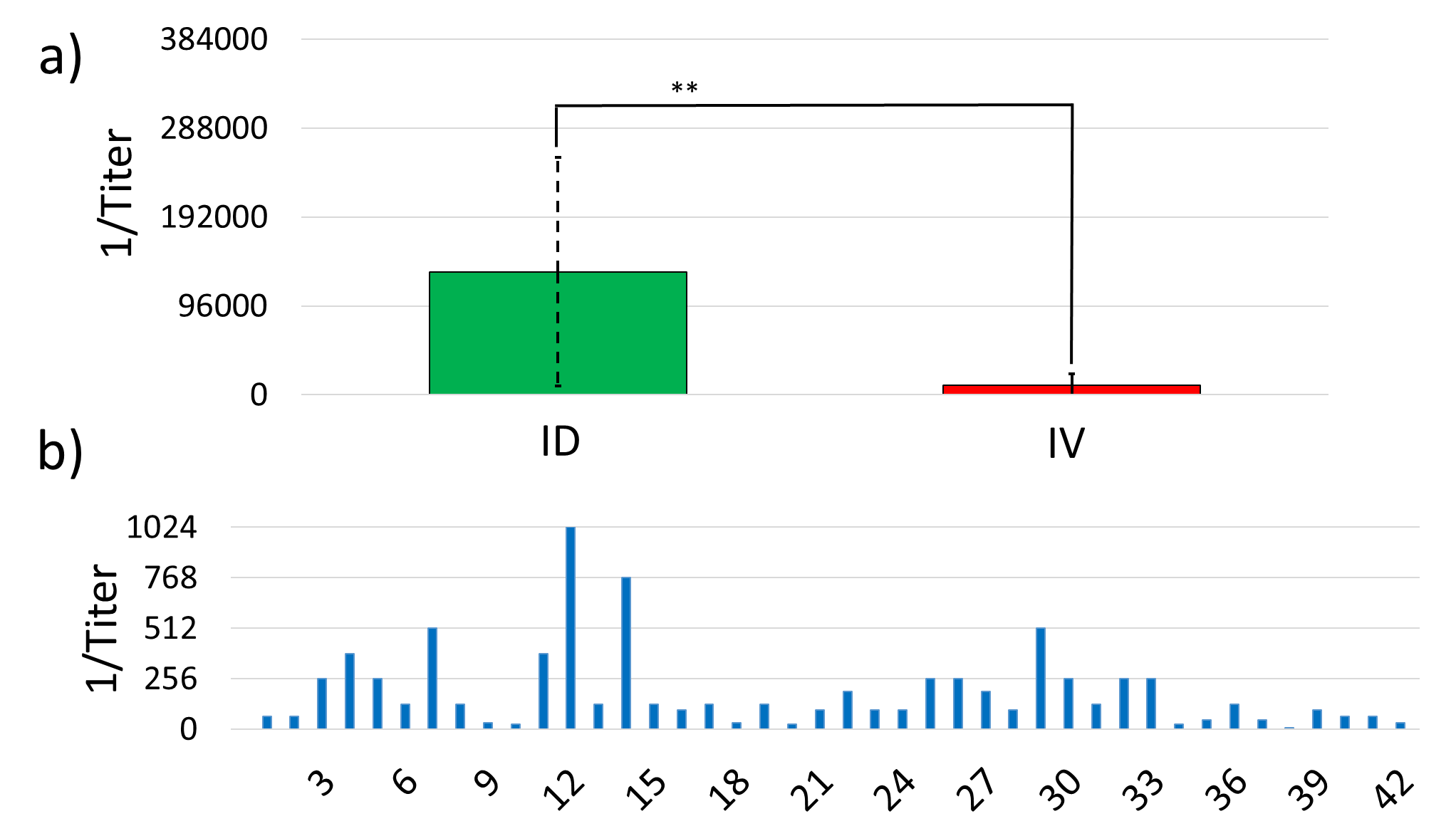
**

**Figure S2b. Analysis of variance (ANOVA) of the titer values** (expressed as 1/titer ± standard deviation) of the groups of rabbits immunised by intradermal route (ID, n=20) and intravenous route (IV, n=20), showing higher average titre values, originating from ID injections (a)**.** Graphical representation of 1/titre values of the 42 sera obtained by ID route (b). ** = p < 0.01.

**Supporting Information S3: Signal background and Technical reproducibility of the indirect colorimetric ELISAs used for titer and specificity assessments.**

1. **Signal background of the secondary antibody (goat anti rabbit peroxidase conjugate) against different strains:** the colorimetric signal generation due to the enzyme reaction spans from 1% (0.042 OD units in an empty well) to 100% (4 OD units) of the limit after which deviation from linearity occurs in the optical detections system (4-6 OD units). In relation to the background signals (those originating independently of the presence of the primary antibody or of the coating) the signal generation is around 0.05 -0.07 OD units (nonspecific background on non-coated wells, see NSB values in Figure S3a-β, intersections G7 and H7), and around 0.042-0.22 OD units (nonspecific background on coated wells, see the values in Figure S3a-γ), depending on the coating (empty well or specific strain, see Figure S3a-α).

**
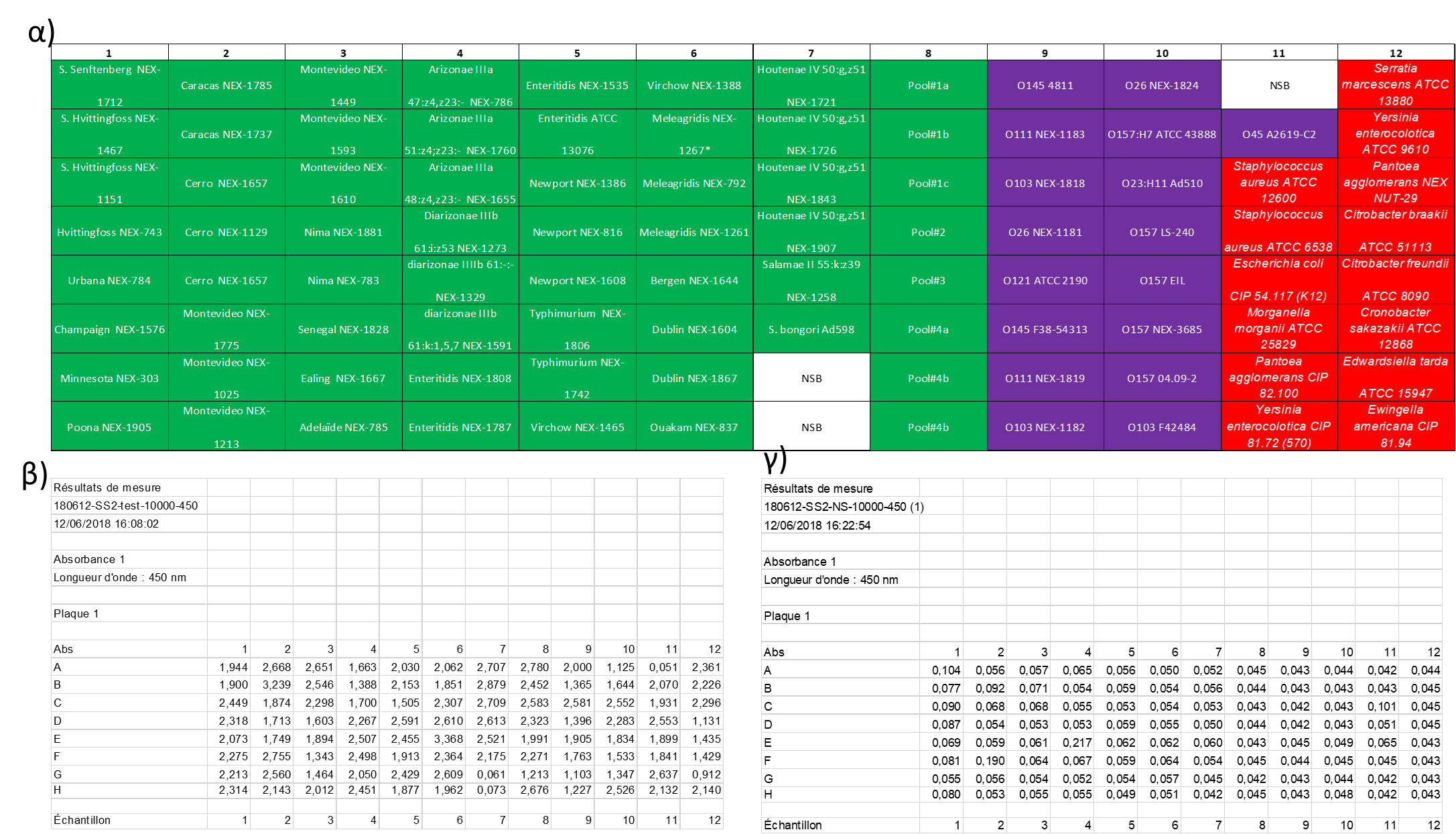
**

**Figure S3a.** Colorimetric responses (*CRs*) of wells coated with both sets of Inclusivity (*Salmonella,* green cells) and exclusivity strains (*E. coli,* purple cells and other strains, red cells), which in a typical experiment range from 0.06 (nonspecific binding of the goat anti rabbit HRP conjugate against the goat plasma saturation, interceptions G7, H7 and A11 in this plate) to less than 4 units of optical density in the presence of anti *Salmonella* sera. At the bottom right, the same ELISA but without the primary antibody (anti *Salmonella* sera), to reveal the nonspecific binding of coated wells, which ranges between 0.042 (the same value obtained for wells without any coating/liquid) to 0.104 (around 3% of the maximal CR of the ELISA obtained with the primary antibody).

1. **Signal background of the primary antibodies (the different sera) against an irrelevant protein**

The values of the dilution-dependent colorimetric responses of the 42 sera against plates coated with insulin are reported in Figure S3b. In every rectangle, the values on the top represent the 1/dilution values, while at the bottom one can find the corresponding colorimetric responses. As an example, serum *1* was evaluated with dilutions ranging from 1/4,000 to 1/512,000, yielding *CRs* ranging from 0.207 to 0.112. In the specificity experiments, it was used at a dilution of 1/16,000, which yields a corresponding background of 0.119.

**Figure S3b.** Colorimetric responses of the 42 sera against plates coated with an irrelevant protein (insulin, 1 µg/mL, 100 µL/well, see Methods for the coating buffer description). Values are colour coded basing on the highest value (1.087, plate#4, serum *37*, dilution 1/3,000, red colour) to the lowest one (0.049, plate#4, serum *41*, dilution 1/64,000, green colour). Those values underlined, italicised and bolded are related to the α value (the dilution that was employed for specificity assessment). Two values from the same column mean that the dilution used for specificity assessments falls between the two values.

The values range from 0.06 (serum *15*) to 0.376 (serum *34*) with a mean of 0.13±0.07 (Figure S3c). The sera yielding colorimetric responses between 0.2 – 0.3 units are *20*, *34*, *36*, *37*, *38*, thus suggesting that the background related to sera properties is irrelevant after 0.3 units. This background is much more than the one originating from the secondary antibody only (around 0.042-0.104, see Figure S3a), which is few units more than what results from the reading of empty wells (0.044). Since the maximal colorimetric response (*MR*) is in the range 2.5-3.5 OD units, the worst background (serum *34*) may account for around 10%-15% of such signal. It should be pointed out that serum *34* is a low titer, pretty aspecific serum, which was not used further for mixture optimization.

**Figure S3c.** Colorimetric responses of the 42 sera at the dilution used for specificity assessment as reported in Figure 1 in the body text. Two values from the same row mean that the dilution used for specificity assessments falls between the two values.

1. **Technical reproducibility of two different operators during independent ELISA on the same set of sera.**

The individual blood withdrawals (BW1-4, corresponding to the samplings occurred at day 38, 66, 94 and 122) composing the two sera *37* and *38* were analysed upon two-fold dilutions from 1/1,000 to 1/128,000 by colorimetric indirect ELISA, to determine the titer values (dilution that mostly approach 1 unit of optical density at 450 nm). The determinations were carried out by two different operators (1 and 2) in two different days but starting from the same 1/10 dilution of the antisera.

The graphs show very similar colorimetric results, and an accuracy in attributing the titer of the individual BWs of ± 1 dilution value (worst-case scenario).

Indeed, for *37* both operators determine titers of 1/8,000, 1/32,000, 1/64,000, and 1/96,000 to the four BWs, while for *38* there is a difference of “half dilution” (BW#3: 1/16,000 or 1/24,000) and of one dilution (BW#4: 1/12,000 or 1/24,000) between the two determinations.


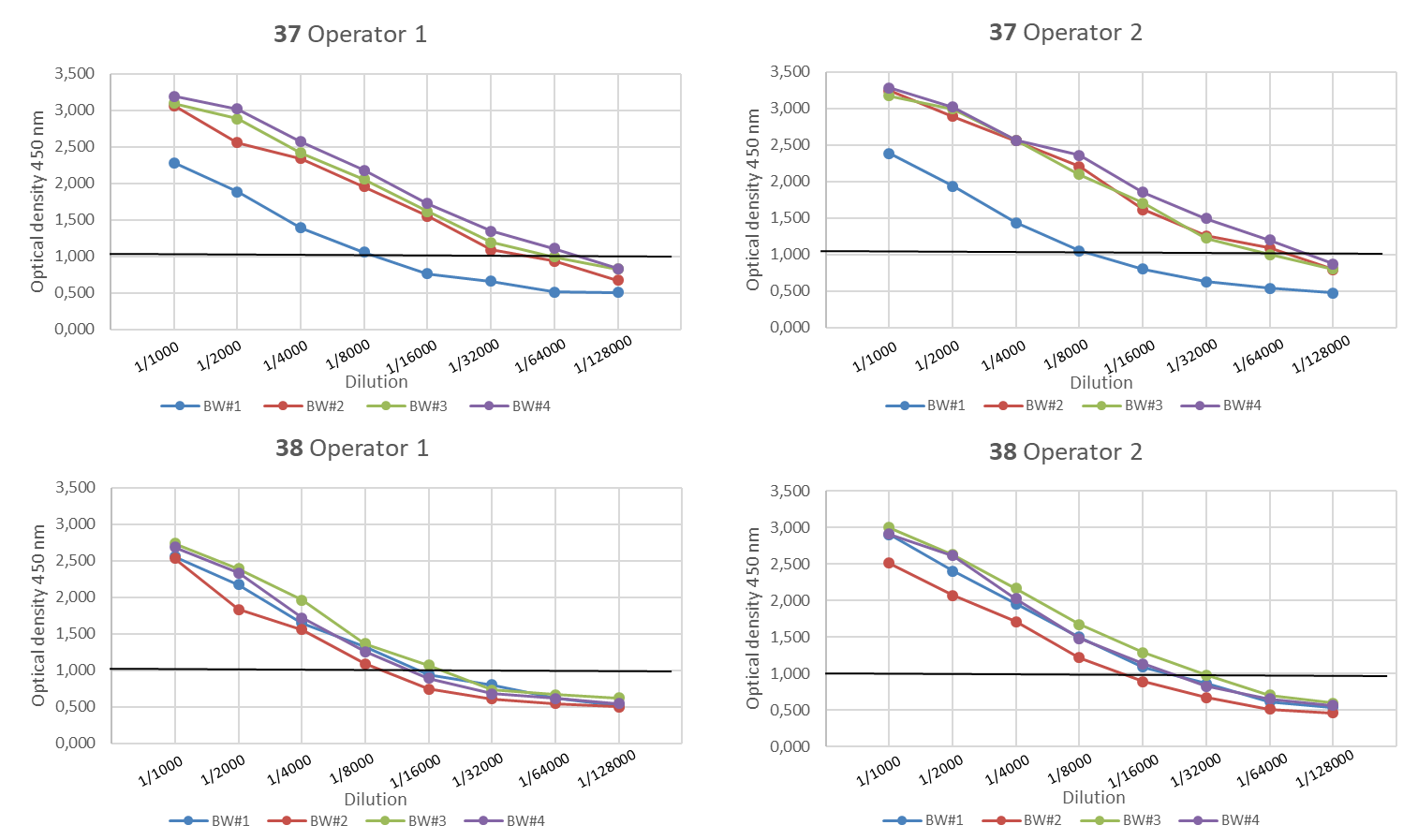


**Figure S3d.** Dilution-dependent optical density plots allowing for titer determination (dilution that yields an optical density of 1) on two sera (*37* and *38*) during assays performed by two different operators.

Once pooled together, the four BWs were again assessed for titer determination 1/48,000 and 1/8,000 (for sera *37* and *38***,** respectively).

1. **Repetition of specificity determination (inclusivity assay) during different days, but by the same operator.**

At two different days, an inclusivity assay on an ensemble of concentrated sera (MIX#4, see the section 2.c Specificity harmonization of serum mixtures in the body text) yield the following results, which indicate inter-assay coefficients of variation for each *NR%* ranging from 0% to 20% (mean of the CV% = 7%±5%).

**
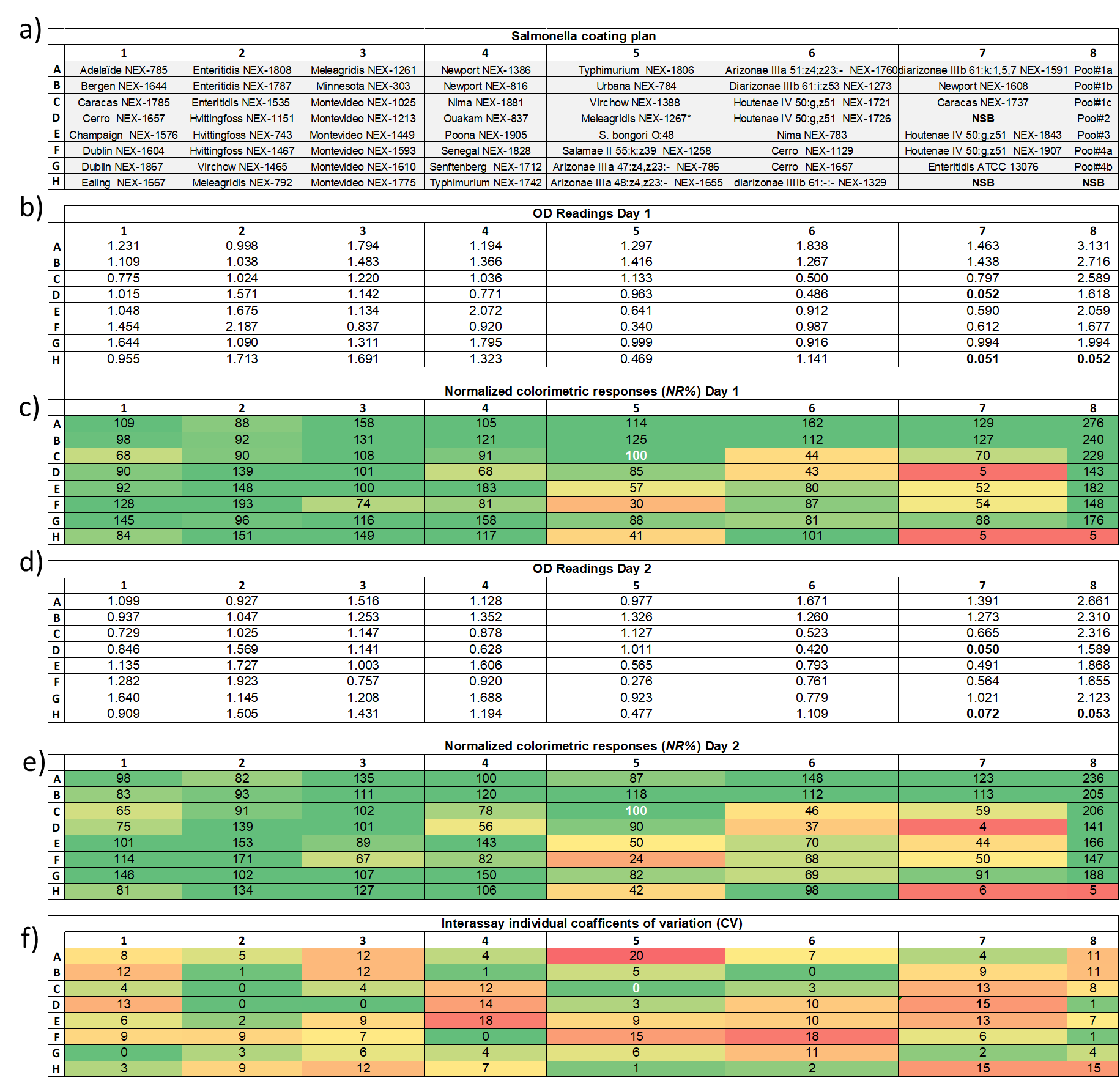
**

**Figure S3e.** Position-dependent (A-H, 1-8) strain coating (a), colorimetric response during the assay performed at different days (day 1, b) or day 2, d)) and normalized colorimetric response (*NR%,* c,e*)* of the dataset against *S. Virchow* NEX-1388, a strain commonly recognized by different anti *Salmonella* sera. Inter-assay coefficients of variation % (CV%) are reported in dataset f).

1. **Specificity determination (inclusivity assay) on MIX#4 (as antisera or as purified IgG fraction).**

At first, we isolated the total IgG fraction of MIX#4 prepared by concentrated sera, by Fast Protein Liquid Chromatography (FPLC) using a Protein A column (HiTrap Protein A from GE Life Sciences) on an AKTA M25 apparatus. FPLC chromatogram (see below) reports an Area of 85% for the peak that flows through the column (mixture of serum proteins, Peak A) and 15% for the peak that eluted upon column acidification (total IgG fraction, Peak B).


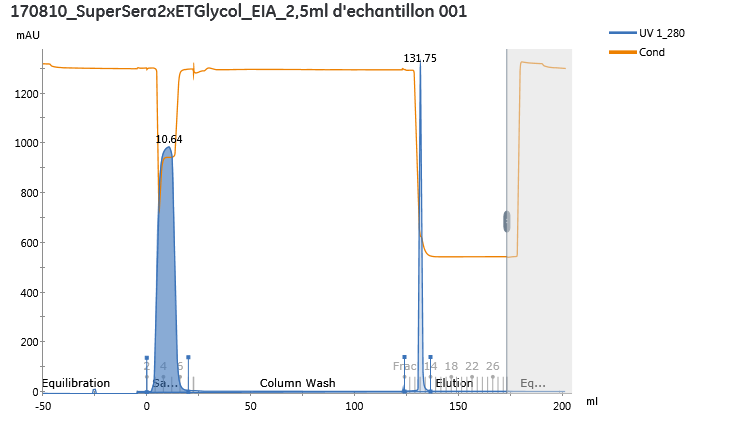


**Figure S3f.** FPLC chromatogram after separation of MIX#4_(prep.)_ constituents by Protein A*.* The trace reports the absorbance at 280 nm as a function of the volume of solvent used, and highlights the presence of a majority peak (serum constituents that pass-through the column during the sample application step) and the IgG fraction, which is retained and subsequently eluted by a pH change step.

The protein concentration of MIX#4, PEAK A and B was then estimated by the Bicinchoninic acid assay (Pierce), using Bovine Serum Albumin Standard Pre-Diluted Set (Pierce 23208) for the external calibration curve.

The concentrations resulted as 83 mg/mL for MIX#4, 1.9 mg/mL for PEAK A, and 1.6 mg/mL for Peak B. These estimated concentrations and IgG/ total protein ratio allowed to define the dilutions of Peak A and B necessary to perform a fair comparison between the specificity of MIX#4 used as a serum and its IgG fraction (IgG-MIX#4). The results are reported below and show a variation of the *NR%* in the range of 0%-19% (mean of the CV% = 6%±5%).


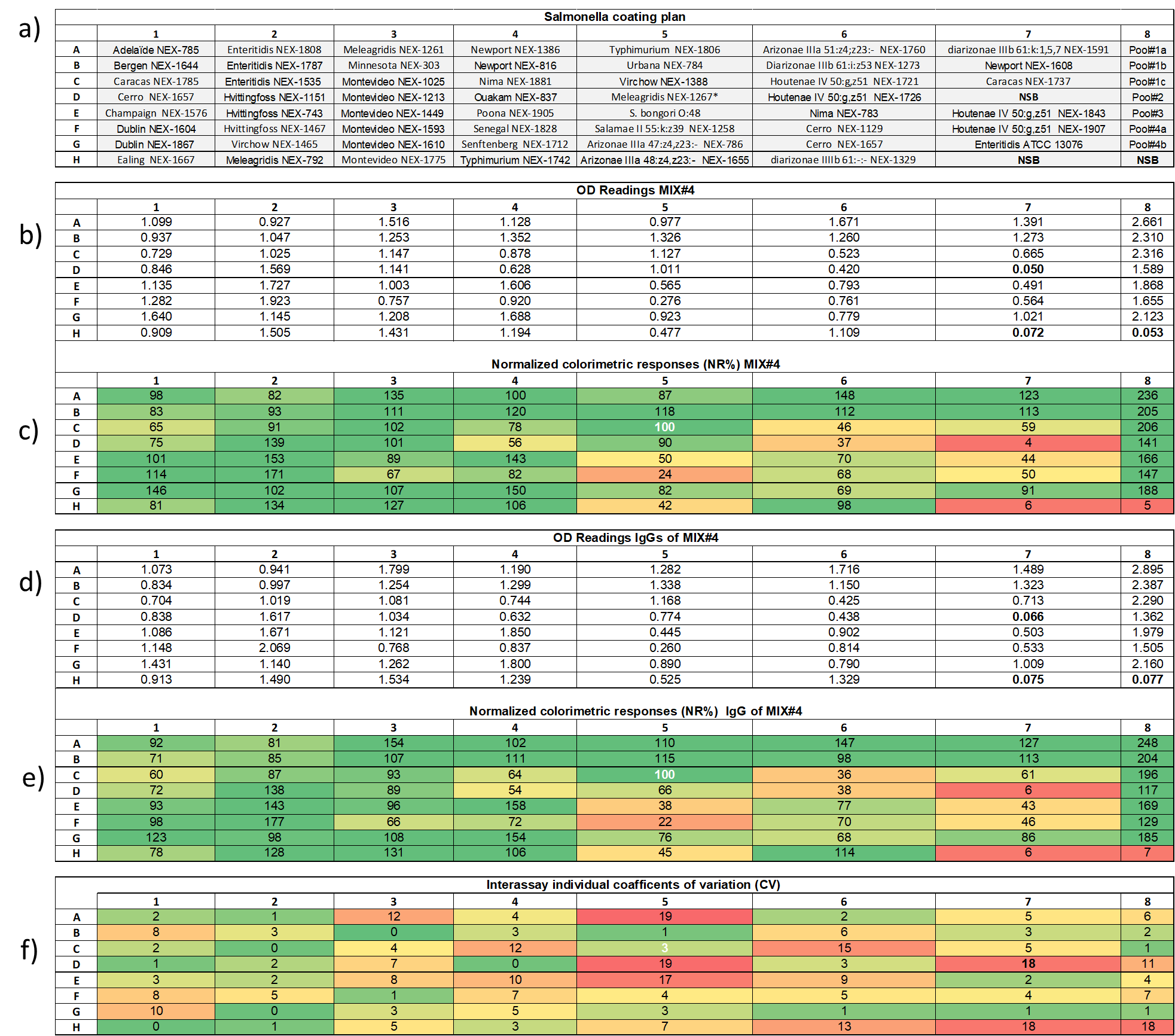


**Figure S3g.** Position-dependent (A-H, 1-8) strain coating (a), colorimetric response during the assay performed with whole MIX#4_(prep.)_ (b) or its IgG fraction (d) and normalized colorimetric response (*NR%,* c,e*)* of the dataset against *S. Virchow* NEX-1388, a strain commonly recognized by different anti *Salmonella* sera. Interassay coefficients of variation % (CV) are reported in dataset f).

**Supporting Information S4: ANOVA of the *NR%* dataset in Figure 1, theoretical *NR%* distribution, and ANOVA of the α values.**

**
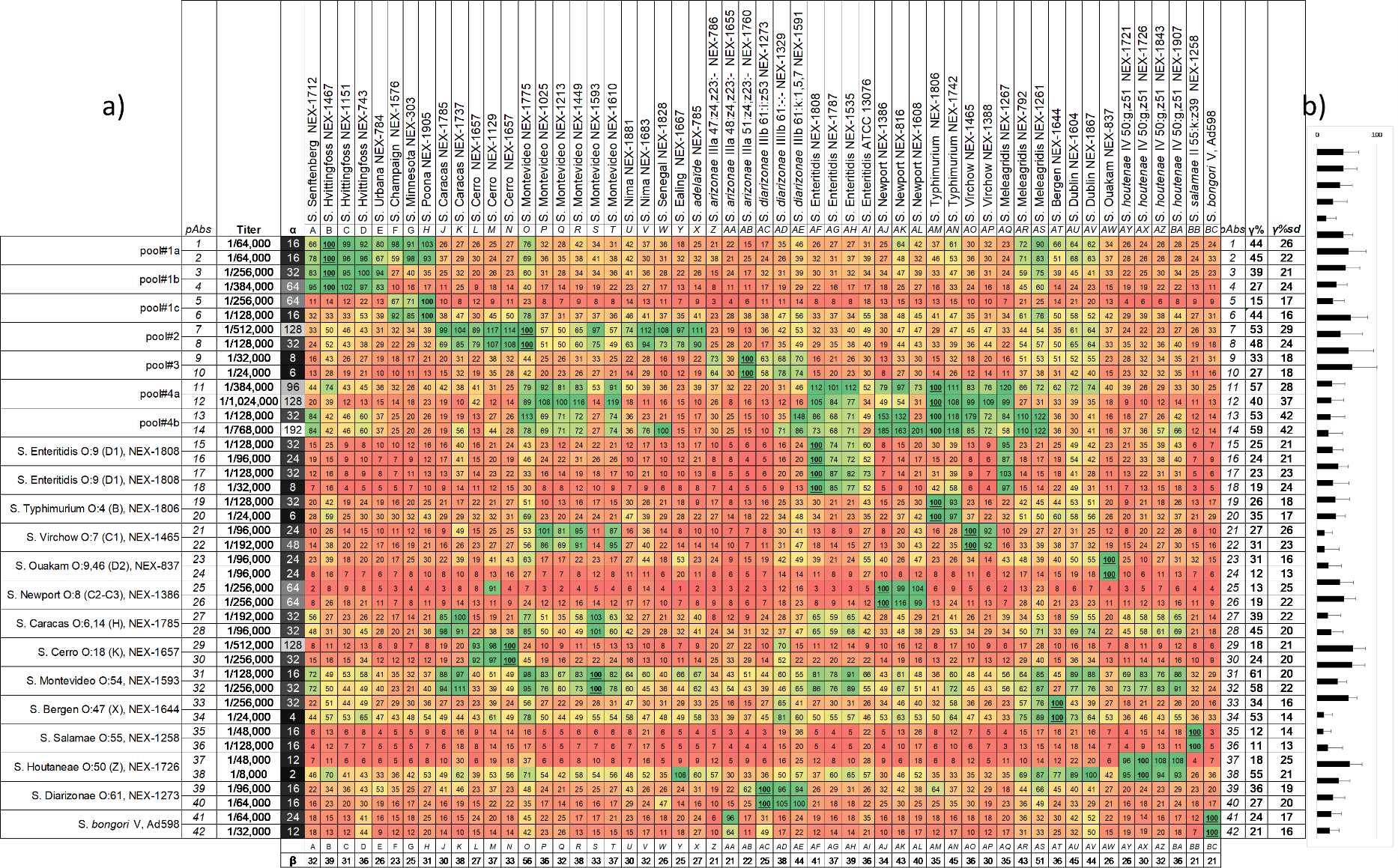
**

**Figure S4a. Specificity heatmap of anti *Salmonella* sera** (Figure 1 in the body text). The normalized colorimetric responses (*NRs%*) of 42 sera (rows 1-42) against the 54 *Salmonella* strains (columns A-BC) are shown. The second column reports the dilutions emerged from titer assessment, while the third one those used for the specificity assessment (the *α* values), which are colour coded from black (low *α* values) to white (high *α* values). At the interceptions between the serum and its immunogen, the colorimetric response is normally the maximal ones (*MR*), and is set as 100 (*NR%*, e.g. see the interceptions 41-BC and 42-BC for the two sera directed against S. *bongori* V, Ad598), while all the other *NRs%* range between 1 and 100. The *NRs%* are colour-coded from red (towards 0) to yellow (50), to green (100). The bottom row reports the β% values for every strain, while the two rightmost columns show the ***γ%±γ%_SD_*** for every serum (see section 2.b).

**Table S4-a.** Dataset emerging from the one-way ANOVA (p<0.05) of the “Scenario A”**,** namely 42 levels (the sera) and the 54 values of colorimetric responses they yield (*NR%*). The standard deviation for the group is 22.68.

**Table S4-b.** Dataset emerging from the one-way ANOVA (p<0.05) of the “Scenario B”**,** namely the 21 levels (the immunogens used) and the 54 values of colorimetric responses they yield (*NR%* values in Figure 1). The standard deviation for the group is 21.89.

**Table S4-c.** Multiple pairwise comparisons (Tukey, p<0.05) of the ANOVA reported in Table S4-a. The sera that does not share any letter have significantly different (p<0.05) means. For instance, sera *31* and *14* (entri#1-2) have a greater means than *12* (entry#14), while *24*, *25*, *35* and *36* (entry#39-42) have smaller means than serum *23* (entry#21). For a description of the meaning of γ%, please refer to the section “sera ranking by descriptors” in the body text.

**Table S4-d.** Multiple pairwise comparisons (Tukey, p<0.05) of the ANOVA reported in Table S4-b. The sera that does not share any letter have significantly different (p<0.05) means. For instance, the immunogen used for the biological replicates *31-32* determine greater means than the immunogen used for the biological replicates *1-2*, *33-34…*, while the immunogen used for the biological replicates *35-36* determine smaller means than the immunogen used for the biological replicates 21-22. For a description of the meaning of γ%, please refer to the section “sera ranking by descriptors” in the body text.

**Table S4-e.** mean α values of the biological replicates originating from the 21 immunogens, allowing the calculation of the standard deviation (SD) and the coefficient of variation % (CV%). ANOVA of the 42 α values originating these means does not reveal statistically significant differences after pairwise comparisons (Tukey, p<0.05) probably due to the large CV% observed for some immunogens, due to the biological replicates variability (e.g. sera *13*-*14*, *19*-*20*, *23*-*24*…).

**Figure S4b.** Specificity heatmap of a library of “imaginary” sera, showing responses of either 100% or 0% to an increasing number of strains. From this dataset, the theoretical limits of the **γ%**±**γ%_SD_** distribution are reported in Figure 2c. The colour code for the colorimetric response is red (values = 0%), and green (values = 100%). At the first row, a sera showing 1 response of 100% and 53 responses of 0%. At the second row, two responses of 100% and 52 responses of 0% and so on and so forth up to the last row, with a sera showing 54 responses of 100%.

**Supporting Information S**5**: On the ranking by the δ descriptor.**

1. **Definition of the δ descriptor**

To create a descriptor that account for both α and γ% values, the simplest way is to create an equation such as δ = α X γ% X C, where C is 1/10,000 (to reduce the magnitude of the δ value and keep it to the range of both α and γ% values). Table S5-a reports the δ values obtained by such equation, where the power of γ% is 1. One can notice that sera having reciprocal values of α and γ% (*e.g.* sera *5* and *31* or sera *26* and *27*).

**Table S5a.** δ values resulting from the equation δ = α X γ% X 0.0001.

| sera | titer | α | **γ%** | δ |
| --- | --- | --- | --- | --- |
| *14* | 1/768000 | 192 | 59 | 113 |
| *7* | 1/512000 | 128 | 53 | 68 |
| *11* | 1/384000 | 96 | 57 | 54 |
| *12* | 1/1024000 | 128 | 40 | 51 |
| *29* | 1/512000 | 128 | 18 | 23 |
| *32* | 1/256000 | 32 | 58 | 19 |
| *4* | 1/384000 | 64 | 27 | 17 |
| *13* | 1/128000 | 32 | 53 | 17 |
| *8* | 1/128000 | 32 | 48 | 15 |
| *22* | 1/192000 | 48 | 31 | 15 |
| *28* | 1/96000 | 32 | 45 | 14 |
| *3* | 1/256000 | 32 | 39 | 12 |
| *27* | 1/192000 | 32 | 39 | 12 |
| *26* | 1/256000 | 64 | 19 | 12 |
| *33* | 1/256000 | 32 | 34 | 11 |
| *31* | 1/128000 | 16 | 61 | 10 |
| *5* | 1/256000 | 64 | 15 | 10 |
| *19* | 1/128000 | 32 | 26 | 8 |
| *15* | 1/128000 | 32 | 25 | 8 |
| *25* | 1/256000 | 64 | 13 | 8 |
| *30* | 1/256000 | 32 | 24 | 8 |

To increase the contribution of γ% in to the equation, a simple way is to create an equation such as δ = α X (γ%)^2^ X C (where C is 1/10,000, to reduce the magnitude of the δ value and keep it to the range of both α and γ% values). Table S5-b reports the δ values obtained by such equation, where the power of γ% is 2. One can notice that sera having reciprocal values of α and γ% (*e.g.* sera *5* and *31* or sera *3* and *27*) shows now a different ranking compared to the one reported in table S5-a..

**Table S5b.** δ values resulting from the equation δ = α X γ%**^2^** X 0.0001.

| sera | titer | α | **γ%** | δ |
| --- | --- | --- | --- | --- |
| *14* | 1/768000 | 192 | 59 | 66 |
| *7* | 1/512000 | 128 | 53 | 36 |
| *11* | 1/384000 | 96 | 57 | 31 |
| *12* | 1/1024000 | 128 | 40 | 21 |
| *32* | 1/256000 | 32 | 58 | 11 |
| *13* | 1/128000 | 32 | 53 | 9 |
| *8* | 1/128000 | 32 | 48 | 7 |
| *28* | 1/96000 | 32 | 45 | 6 |
| *31* | 1/128000 | 16 | 61 | 6 |
| *3* | 1/256000 | 32 | 39 | 5 |
| *27* | 1/192000 | 32 | 39 | 5 |
| *22* | 1/192000 | 48 | 31 | 5 |
| *4* | 1/384000 | 64 | 27 | 5 |
| *29* | 1/512000 | 128 | 18 | 4 |
| *33* | 1/256000 | 32 | 34 | 4 |
| *2* | 1/64000 | 16 | 45 | 3 |
| *6* | 1/128000 | 16 | 44 | 3 |
| *1* | 1/64000 | 16 | 44 | 3 |
| *23* | 1/96000 | 24 | 31 | 2 |
| *26* | 1/256000 | 64 | 19 | 2 |
| *19* | 1/128000 | 32 | 26 | 2 |

To further increase the contribution of γ% in to the equation, a simple way is to create an equation such as δ = α X (γ%)^3^ X C (where C is 1/10,000, to reduce the magnitude of the δ value and keep it to the range of both α and γ% values). Table S5-c reports the δ values obtained by such equation, where the power of γ% is 3. One can notice that sera having reciprocal values of α and γ% (*e.g.* sera *5* and *31* or sera *3* and *27*) that were equally ranked in table S5-a and differently ranked in table S5-b are no longer in the table.

**Table S5c.** δ values resulting from the equation δ = α X γ%**^3^** X 0.0001.

| sera | titer | α | **γ%** | δ |
| --- | --- | --- | --- | --- |
| *14* | 1/768000 | 192 | 59 | 389 |
| *7* | 1/512000 | 128 | 53 | 193 |
| *11* | 1/384000 | 96 | 57 | 174 |
| *12* | 1/1024000 | 128 | 40 | 82 |
| *32* | 1/256000 | 32 | 58 | 64 |
| *13* | 1/128000 | 32 | 53 | 48 |
| *31* | 1/128000 | 16 | 61 | 36 |
| *8* | 1/128000 | 32 | 48 | 36 |
| *28* | 1/96000 | 32 | 45 | 29 |
| *3* | 1/256000 | 32 | 39 | 19 |
| *27* | 1/192000 | 32 | 39 | 19 |
| *22* | 1/192000 | 48 | 31 | 14 |
| *2* | 1/64000 | 16 | 45 | 14 |
| *6* | 1/128000 | 16 | 44 | 14 |
| *1* | 1/64000 | 16 | 44 | 14 |
| *33* | 1/256000 | 32 | 34 | 13 |
| *4* | 1/384000 | 64 | 27 | 13 |
| *39* | 1/96000 | 16 | 36 | 8 |
| *29* | 1/512000 | 128 | 18 | 8 |
| *23* | 1/96000 | 24 | 31 | 7 |
| *34* | 1/24000 | 4 | 53 | 6 |

1. **Descriptors values variability based on background signals and technical reproducibility**

Technical replication of titer determination resulted reproducible (same titers determination in 6 out of 8 instances, then variation of “half dilution” and one dilution, at most). Therefore, there is a “worst case scenario” where a reader attempting to reproduce the results (or adapt to its own case) will have a variation of either “half-dilution” or one dilution for each of the tested sera.

In the same way, technical replication of the specificity profiles shows a mean for the coefficients of variation of 6%±5%, with few instances showing up to 20%.

Let us consider a hypothetic scenario, where all of the colorimetric responses of every sera vary of ±10%. This will mean that the specificity heatmap as reported in Figure 1 (simplified here to the 21 best biological replicates) could yield multiple combinations of colorimetric responses, but with two extreme boundaries:

The case where all of the colorimetric responses result 10% lower than what we have determined.

The case where all of the colorimetric responses result 10% higher than what we have determined.

Let us now combine these two scenarios (variance in titer of ± “half dilution” and of ±10% of colorimetric responses during specificity), to verify whether the resulting **δ** values will yield populations that can still be differentiated by statistical means.

The nine combinations are:

**δ1:** with every α used at α minus “half dilution” and every γ% calculated by reducing by 10% every colorimetric response compared to the *NR%*.

**δ2:** with every α used at the value we have experimentally determined and every γ% calculated by reducing by 10% every colorimetric response compared to the *NR%*.

**δ3:** with every α used at α plus “half dilution” and every γ% calculated by reducing by 10% every colorimetric response compared to the *NR%*.

**δ4:** with every α used at α minus “half dilution” and the γ% we have experimentally determined.

**δ5:** with every α used at the value we have experimentally determined and the γ% we have experimentally determined.

**δ6:** with every α used at α plus “half dilution” and the γ% we have experimentally determined.

**δ7:** with every α used at α plus “half dilution” and every γ% calculated by increasing by 10% every colorimetric response compared to the *NR%*.

**δ8:** with every α used at the value we have experimentally determined and every γ% calculated by increasing by 10% every colorimetric response compared to the *NR%*.

**δ9:** with every α used at α + “half dilution” and every γ% calculated by increasing by 10% every colorimetric response compared to the *NR%*.

δ is calculated according to the following equation, and the resulting computations are summarized in Table S3, which report for the sera one real data (the δ we have determined, δ2 in this table) and 8 simulated data resulting from the combinations of the aforementioned variances of titer (± “half dilution”) and specificity (±10%).

**δ= α γ^3/100,000**

ANOVA (p<0.05) of the factor “sera” with the 9 δ values as response, and pairwise comparison (Tukey, p<0.05, Table S3a), still allows highlighting the sera that show the best combination of titer and specificity (14, 7 and 12).

If we consider other scenarios with more variance (e.g. variance of titer of ± “half dilution” and specificity variation of ± 20%, or variance of titer of ± 1 dilution and specificity variation of ± 10%) the ANOVA still highlights sera 14 and 7 (but no longer 12). We thus suggest the interested reader to verify the technical reproducibility in titer determination and specificity assessment prior to the execution of this approach, to know in advance which is the degree of variance of its own system, and thus how far can him infer from his dataset.

**Table S5d. Pairwise comparison (Tukey, p< 0.05). Means not sharing any letters are significantly different.**

| **serum** | **N** | **Mean** | **Group** | | | |
| --- | --- | --- | --- | --- | --- | --- |
| 14 | 9 | 428.6 | A |  |  |  |
| 7 | 9 | 212.9 |  | B |  |  |
| 12 | 9 | 90.5 |  |  | C |  |
| 32 | 9 | 70.26 |  |  | C | D |
| 28 | 9 | 32.19 |  |  | C | D |
| 3 | 9 | 20.76 |  |  | C | D |
| 22 | 9 | 15.94 |  |  |  | D |
| 2 | 9 | 15.56 |  |  |  | D |
| 6 | 9 | 15.31 |  |  |  | D |
| 33 | 9 | 14.23 |  |  |  | D |
| 39 | 9 | 8.49 |  |  |  | D |
| 29 | 9 | 8.47 |  |  |  | D |
| 23 | 9 | 8.26 |  |  |  | D |
| 19 | 9 | 6.500 |  |  |  | D |
| 15 | 9 | 5.829 |  |  |  | D |
| 26 | 9 | 4.892 |  |  |  | D |
| 17 | 9 | 4.418 |  |  |  | D |
| 41 | 9 | 3.835 |  |  |  | D |
| 38 | 9 | 3.578 |  |  |  | D |
| 9 | 9 | 3.120 |  |  |  | D |
| 35 | 9 | 0.2896 |  |  |  | D |

**Supporting Information S6: serum mixtures (MIXs) composition and specificity.**

**Figure S6a:** **MIXs** composition matrix, showing the sera used, their α value, and their immunogen properties (species, subspecies, serovars, O Group, strain). Red colour highlights the limiting reagent (sera) for the preparation of the **MIX,** (e.g. sera *37* with an α of 12, for **MIXs#1**).

**Table S6a**. Pairwise comparison after ANOVA (Tukey, p< 0.05) of specificity datasets (inclusivity, see Figure S6b) of some of the best individual sera (*12* and *14*) and the mixtures 1-4, along with the reference from KPL. Means not sharing any letters are significantly different.

**Figure S6b.** Predicted (artificial *β%*) and experimental (from diluted sera or concentrated sera) specificity heatmaps of MIXs inclusivity, showing the normalized colorimetric responses (*NR%*) of four MIXs against the *Salmonella* strains, by colour coding from red (*NR%* values towards 0), yellow (towards 50), and green (towards 100).

**Table S6b**. Pairwise comparison after ANOVA (Tukey, p< 0.05) of specificity datasets (inclusivity) of some of mixtures 1-4, along with the predicted values (pred.). “conc.” Means the realisation of a mixture directly from serum rather than from its dilutions. Means not sharing any letters are significantly different. It can be seen that the datasets resulting from “predicted”, “experimental” or “concentrated” mixtures are indistinguishable on a “per MIX” basis.

**Figure S6c.** Inclusivity (top) and exclusivity (down) heatmaps, showing the normalized colorimetric responses (*NR%*) of the sera and MIXs depicted in Figure 3d, colour coded from red (*NR%* values towards 0), yellow (towards 50), and green (towards 100).

**Table S6c. Pairwise comparison after ANOVA (Tukey, p< 0.05) of specificity datasets (inclusivity). Means not sharing any letters are significantly different.**

**Table S6d. Pairwise comparison after ANOVA (Tukey, p< 0.001) of specificity datasets (inclusivity). Means not sharing any letters are significantly different.**

**Supporting Information S7: anti-*Salmonella* sera cross-reactivity towards selected strains from the “exclusivity” set.**

**Table S7a. Pairwise comparison after ANOVA (Tukey, p< 0.05) of specificity datasets (exclusivity against *E. coli*). The α value at which MIXs were used is reported in parenthesis. Means not sharing any letters are significantly different.**

**Figure S7a:** Colorimetric response (optical density) of anti *Salmonella* sera against common strains that can be found in food commodities, showing different levels of responses according to the sera used. Compared to all of the other heatmaps, the colour code for the colorimetric response is red (values towards OD = 0), yellow (values towards OD = 1) and green (values towards OD = 2). It can be easily seen that sera **19**, **21**, **24**, **17** and **28** are those with the highest responses, resulting the least exclusive ones.

**Figure S7b.** Exclusivity heatmap (belonging to the same experiment yielding Figure S6c), showing the normalized colorimetric responses (*NR%*) of the sera and MIXs depicted in Figure 3d, colour coded from red (*NR%* values towards 0), yellow (towards 50), and green (towards 100).

**Table S7b. Pairwise comparison after ANOVA (Tukey, p< 0.05) of specificity datasets (inclusivity). Means not sharing any letters are significantly different.**

**Table S7c. Pairwise comparison after ANOVA (Tukey, p< 0.001) of specificity datasets (inclusivity). Means not sharing any letters are significantly different.**

**Supporting Information S8: Normalized colorimetric responses of plates seeded by the DoE approach for the full combinatorial exploration of three sera at four dilutions.**


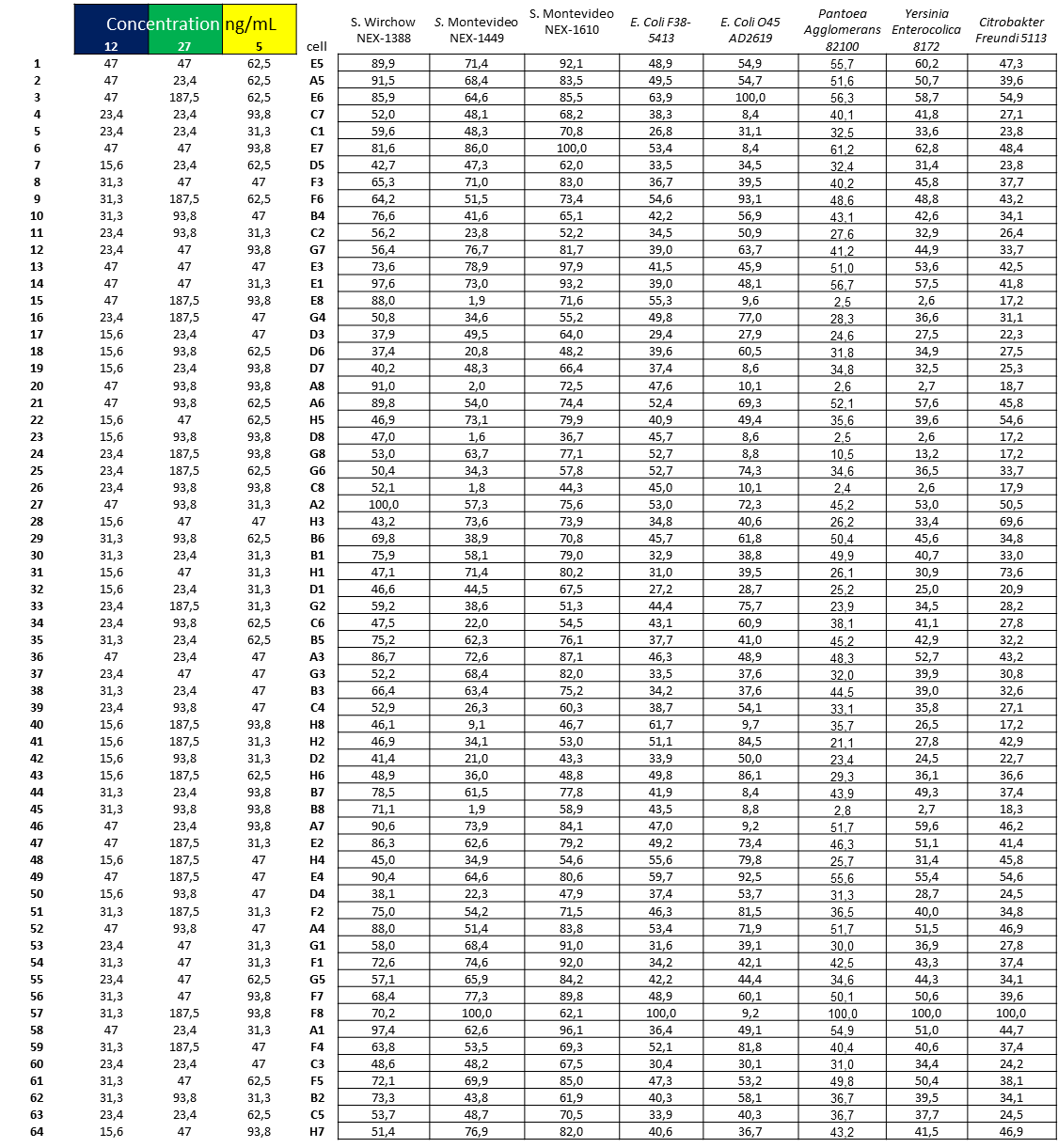


**Figure S8a:** List of the 64 combinations of sera dilutions (expressed as concentrations ng/mL) depicted in Figure 4a, along with the percentage of the colorimetric responses as seen in Figure 4b.


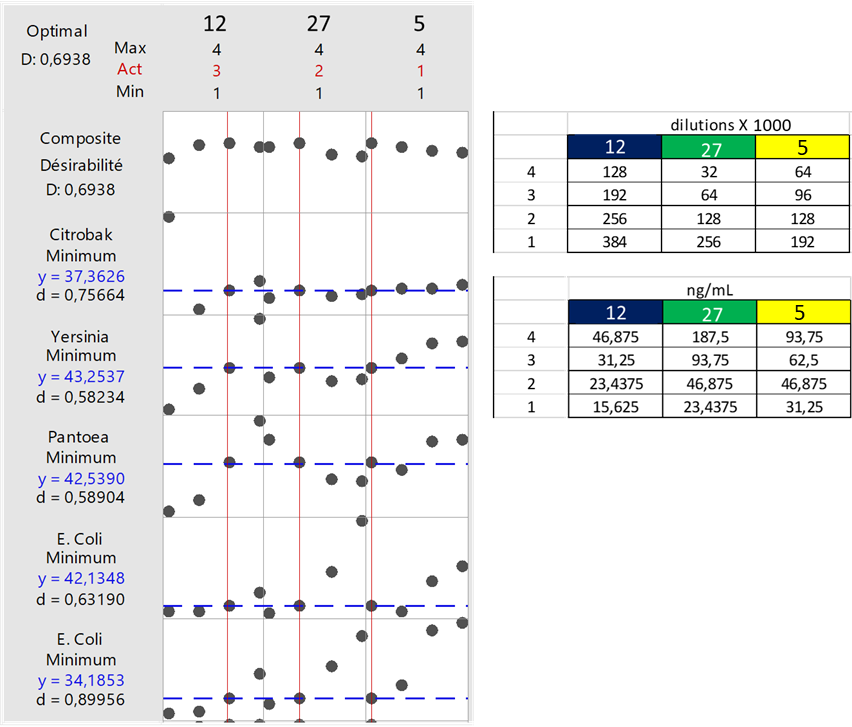


**Figure S8b:** Results of the sDoE using the experimental values reported in Figure S7a. By selecting the objectives of the sDoE (find the best combination to minimize the response against *Y. Enterocolica, P. Agglomerans*, *E. coli*, and maximize the responses against the *Salmonella*), the combination of the levels “3,2,1”, corresponding to the dilutions of 1/192,000, 1/128,000, and 1/192,000 for serum **12**, **27** and **5**, respectively, was identified.
